# Diagnosis of nontuberculous mycobacterial infections using genomics and artificial intelligence-machine learning approaches: scope, progress and challenges

**DOI:** 10.3389/fmicb.2025.1665685

**Published:** 2025-09-03

**Authors:** Madhan Kumar Murthy, Vivek Kumar Gupta, Anand Prakash Maurya

**Affiliations:** ^1^Department of Immunology, ICMR-National JALMA Institute for Leprosy and Other Mycobacterial Diseases (ICMR-NJIL&OMD), Agra, Uttar Pradesh, India; ^2^Department of Biochemistry, ICMR-National JALMA Institute for Leprosy and Other Mycobacterial Diseases (ICMR-NJIL&OMD), Agra, Uttar Pradesh, India; ^3^Department of Clinical Trials and Implementation Research, ICMR-National JALMA Institute for Leprosy and Other Mycobacterial Diseases (ICMR-NJIL&OMD), Agra, Uttar Pradesh, India

**Keywords:** non tuberculous mycobacteria, diagnosis, genomics, artificial intelligence, machine learning, next generation sequencing, targeted next generation, metagenomic next generation sequencing

## Abstract

The nontuberculous mycobacterial (NTM) infections cause morbidity and mortality in individuals who are immunocompromised and those with lung conditions. The timely diagnosis of NTM infections is thus the need of the hour for appropriate management of the disease. In this context, genomics has played a pivotal role in diagnosis of NTM by targeting various conserved regions which are useful for species identification and diagnosis. Also, the exploring of whole genome of nontuberculous mycobacteria has made species identification easier and has revolutionized the diagnostic landscape of NTM. The refinement of Whole Genome Sequencing (WGS) and the advent of targeted Next Generation Sequencing (tNGS) and metagenomic NGS (mNGS) has helped in bringing down the cost without compromising the quality in NTM diagnostics. The advent of artificial intelligence (AI) technologies has made NTM diagnosis even easier by analyzing complex genomic data and providing faster results. Thus, this comprehensive review discusses the strides made in genomics and AI based approaches in the diagnosis of NTM infections and the way forward for harnessing this potential to the maximum for the benefit of mankind.

## Introduction

While mycobacterial infections seem to be causing alarming increase in morbidity and mortality in immunocompetent individuals, the nontuberculous mycobacterial (NTM) infections deserve importance in having their effect predominantly on immunocompromised individuals or those with lung disease or lung abnormalities and also in immunocompetent individuals. Although NTM are ubiquitous in nature and humans are exposed to them, the diseases caused by them are rare (Honda et al., 2018a). Predominantly, NTMs cause infections in the lung and is referred to as ‘NTM pulmonary disease (NTM-PD)‘and is most commonly found in patients with previous lung diseases and also in those with no history of lung disease. They may cause disease in other extrapulmonary sites like skin, lymph nodes, soft tissues and it can also cause disseminated infections in which case it is referred to as ‘NTM non pulmonary disease (NTM-NPD)’ (Ricotta et al., 2021). The rarity of NTM disease can be attributed to the low to moderate pathogenicity of the NTMs and the immune systems of the host as it is common in immunocompromised and less common in immunocompetent individuals. But, the chronicity of NTM infections causes morbidity and mortality in individuals which makes it an important issue to be addressed. This is truer in the case of *Mycobacterium abscessus complex* (MABC) lung disease, *Mycobacterium avium complex* (MAC) lung disease which is associated with significant mortality rates.

Thus, the accurate diagnosis of NTM infections deserves utmost importance for managing the disease effectively, preventing disease progression and for choosing the suitable treatment strategies. Timely diagnosis by using different methods like clinical, radiological, microbiological can help in distinguishing it from other lung infections and for initiating appropriate treatment. This review highlights the epidemiology of NTM, their diagnostic methods, treatment and prognosis with emphasis on genomics in NTM and the utility of AI in NTM diagnosis with the directions for way forward in NTM diagnosis.

## Epidemiology of NTM infections

### Incidence and prevalence of NTM infections

Reports suggest that, NTM incidence and prevalence have risen in recent years, and has thus been given significant attention. The major issue occurs when potential NTM patients are mistakenly identified as drug-resistant TB. A 2013 study carried out in the United States assessed the prevalence at 13.9 in 100,000 people (van Ingen et al., 2021). Between 2009 and 2014, the overall incidence of NTM pulmonary illness in Germany was approximately 3.3 cases in 100,000 people (Ringshausen et al., 2016). Dohál et al. (2023) reported that, in Slovakia, out of 1,355 NTM-positive cultures, NTM illness was identified in 358 instances (26.4%) (Dohál et al., 2023). Among those over 55, the disease was significantly more common (*p* < 0.0001) (Dohál et al., 2023). Additionally, the average age was considerably greater for women with NTM disease than for men (*p* = 0.0005). About 39.9% of NTM disease cases were ascribed to *M. intracellulare*, and 38.5% to *M. avium* (Dohál et al., 2023). According to a multicentric study conducted in Japan between 2001 and 2009, the rate of NTM pulmonary illness was 10.1 per 100,000 people (Prevots and Marras, 2015). In Korea in 2016, the estimated age-adjusted rate and prevalence were 17.9 and 33.3 in 100,000 population, respectively (Park et al., 2019). According to an Iranian study from 1996 to 2023, the prevalence of NTM was less than 15% (Hazar et al., 2024).

In India, the incidence of NTM ranged from 0.7 to 34%, respectively (Thangavelu et al., 2021). A study conducted in Northern India found that the prevalence was almost 29% (Rath et al., 2025). The rate of NTM was reported to be 8.3% in Delhi and around 7.4% in Chandigarh (Umrao et al., 2016). In Kolkata, the rate of infection in bronchial specimens was approximately 17.4% (Umrao et al., 2016). A study conducted in Vellore found that the frequency of NTM in south India was approximately 0.5% (Thangavelu et al., 2021).

### Mortality and geographic distribution of NTM

Because of high rates of therapy failure and incorrect treatment, NTM-PD has a greater mortality rate than *M. tuberculosis* (MTB) (Pennington et al., 2021). In a study by Harada et al. (2025), NTM mortality data between the years 2000–2022 from 83 countries were analyzed. The authors observed that the mortality rate doubled from 0.36 deaths per 1,000,000 individuals in 2000 to 0.77 deaths per 1,000,000 individuals in 2022 (Harada et al., 2025). They also found that NTM associated deaths occurred in people who were aged ≥65 years. The region that showed the highest mortality rate was the Western Pacific region. But the study predominantly included the European and American countries and only a few countries in the eastern Mediterranean, African, South east Asian region because of the absence of data or poor quality of the reported data. The study results highlight the rising mortality rates and vulnerability of the geriatric population to NTM necessitating appropriate diagnosis, treatment and the planning of healthcare (Harada et al., 2025).

Another study found that between the years 2003–2018 in Taiwan, the overall mortality rate due to NTM was 35.2%. The study observed that old age, male gender, use of steroids, anti-neoplastic agents, immunosuppressants, high Charlson comorbidity index were found to be associated with risk of mortality (Chen et al., 2022).

In a study by Pedersen et al. (2024), mortality rate in extrapulmonary NTM infected individuals were studied between 2000 and 2017 in Denmark. Five-year mortality rate of patients was found to be 26.8%. Mortality rate was found to be predominant in those with extrapulmonary NTM infections with underlying comorbidities (neoplasms, cardiovascular diseases, hematologic malignancies, HIV) (Pedersen et al., 2024).

The study by Jhun et al. (2020) assessed the prognostic factors associated with mortality due to NTM infections in South Korea by assessing the data between 1997 to 2013 from NTM registry. The overall cumulative mortality rates for the patients with NTM-PD was 12.4% for 5 years, 24% for 10 years and 36.4% for 15 years (Jhun et al., 2020). Also, the factors like old age, male gender, low body mass index (BMI), chronic heart or liver disease, pulmonary malignancy, chronic pulmonary aspergillosis and erythrocyte sedimentation rate were associated with mortality. The organisms significantly associated with mortality were *M. intracellulare* and *M. abscessus*. The authors also observed that mortality in NTM-PD patients was associated with cavitary forms of the disease (cavitary nodular bronchiectasis and fibrocavitary form) (Jhun et al., 2020).

The geographic distribution of NTM provides details on the factors that contribute to the development of NTM lung disease (NTM-LD)/NTM-PD, which is highly location-specific. The most common NTM in London and the United Kingdom is MAC, which is the most common organism isolated worldwide (van Ingen et al., 2021). In the United States, MAC, *M. kansasii*, and *M. abscessus* from the southeast of the United States were responsible for 80% of lung diseases linked to nontuberculous mycobacteria (To et al., 2020). In Southern Europe, *M. xenopi* was isolated in greater quantities than in Northern Europe (Zulu et al., 2021). The most prevalent species identified in Greece and Canada were *M. lentiflavum* as well as *M. gordonae*, respectively (Zulu et al., 2021). In Australia, *M. intracellulare* was frequently isolated, while *M. abscessus* was frequently seen in Southern Australia (Kalpana et al., 2022; Zulu et al., 2021). Reports of MAC species, primarily *M. intracellulare*, are reported from China. In Taiwan, *M. abscessus* is prevalent in the south and MAC is prevalent in the north. The most prevalent NTMs in Gulf nations are MAC and *M. abscessus* (Prevots and Marras, 2015). In Oman, MAC, *M. simiae*, along with *M. marinum* were regularly seen. In Iran, *M. flavescens* and *M. fortuitum* are more common (Honda et al., 2018b). In Singapore, *M. abscessus* was found to be more prevalent (Prevots and Marras, 2015). In the Indian subcontinent, *M. abscessus*, *M. fortuitum*, and *M. intracellulare* were found to be prevalent (Honda et al., 2018b).

### NTM infections in healthcare settings

The NTM infections in healthcare settings can increase the mortality risk in vulnerable population as well as those with severe underlying health issues. Infections linked to NTM that affect hospitalized patients can range from colonization to epidemics in healthcare settings (Desai and Hurtado, 2018). Between 60 and 100% of hemodialysis units and healthcare facilities have NTM colonization. The use of infected needles and reusable injection equipment has been linked to outbreaks of NTM injection site abscesses (Desai and Hurtado, 2018). There are also reports of infections in NTM cardiac pacemakers and central venous catheters. The transmission of NTM to uninfected individuals occurs when NTM mycobacteria colonize bronchoscope suction valves and channels (Kalpana et al., 2022). The installation of tympanostomy tubes and contaminated otolaryngostomy equipment have resulted in *M. fortuitum* mastoiditis and *M. chelonae* otitis media (Phillips and Von Reyn, 2001). Plastic surgery procedures like lipoaspiration, breast prosthesis insertion, abdominoplasty and other procedures which involve the use of contaminated needles or injection of products contaminated with rapidly growing mycobacteria (RGM) can lead to long-term wounds that are non-healing. The RGM *M. abscessus* and *M. massiliense* are the species that are primarily involved in these infections which manifest as chronic non-healing wounds (Shen et al., 2024; Viana-Niero et al., 2008). Epidemiologically, this deserves importance as the healthy people who neither have a baseline disease nor are immunocompromised (without infections like HIV or other immunocompromising conditions) contract the infections due to cosmetic procedures.

In hospital settings, the disinfectant used for decontaminating medical devices like bronchoscopes, laparoscope and disposable trocars is predominantly glutaraldehyde. Currently, peracetic acid is recommended for disinfection because of its lack of protein-fixation properties unlike glutaraldehyde (Pineau et al., 2008) and its effectiveness against a wide range of microbes like bacteria, viruses as well as spores (Vizcaino-Alcaide and Herruzo-Cabrera, 2003). Also glutaraldehyde is less effective against NTM that are resistant to it whereas peracetic acid is less prone to resistance (Burgess et al., 2017). Earlier studies from different parts of the world have shown that RGM contamination of medical devices or aqueous solutions is implicated in the outbreak of nosocomial infections (Abbas et al., 2024; Desai and Hurtado, 2018; Fontana, 2008).

### NTM prevalence and diagnostics

The diagnosis of NTM infections is challenging because of their omnipresence in the environment (in soil and water resources). Because of this, isolating it from a non-sterile respiratory specimen does not necessarily imply that to be the causative agent of that lung disease (van Ingen, 2015). Also, clinically the presentation of symptoms is not specific and hence it leads to delayed diagnosis. The diagnosis of NTM infections is also influenced to a greater extent by the geographic prevalence. Different regions in the world have various NTM species which lead to various types of infections (NTM-PD or NTM-NPD) which in turn impacts the diagnostic frequency. The distribution of NTM found in the soil and water resources is influenced by multiple factors like climate, water sources, temperature. Thus, some NTM are adapted for thriving in tropical climates while others are found commonly in temperate regions. Few examples of geographic variation in NTM species distribution are the presence of MAC followed by *M. kansasii* (Hoefsloot et al., 2013) in North America, South America and *M. abscessus* in East Asia (Lee M. R. et al., 2015). Multiple factors like age, sex and underlying lung conditions (like bronchiectasis) can impact an individual’s susceptibility to NTM infection and disease. Also, high rates of NTM prevalence are reported in coastal areas when compared to inland areas because of factors like soil composition and water sources (Schildkraut et al., 2020). Thus, if the clinicians are aware of the local NTM epidemiology, it might be useful to guide diagnostics and for administering appropriate treatment.

### AI in the diagnosis of NTM

Clinically NTM diagnosis is challenging because of the presentation of non-specific symptoms and difficulty in isolating the NTM species. Hence the clinical syndrome should be correlated with radiologic diagnosis and then confirmed microbiologically for establishing the diagnosis of an NTM infection in the case of NTM-PD. But, currently, the diagnosis of NTM-PD is made based on the guidelines by American thoracic society (ATS) and infectious disease society of America (IDSA) which specifies the requirement that positive sputum culture should be consistently demonstrated on two different time points and for non-sterile sites (like cephalic and cardiac fluids) when specimens like biopsies and blood will be used, one sample will be sufficient for diagnosis (Daley et al., 2020). In the case of bronchoalveolar lavage (BAL) obtained from lower respiratory tract, one positive sample or one positive BAL (sterile site) is suggested to be sufficient for diagnosis. This sample can be further processed for culture or species identification If the initial tests are not able to diagnose NTM, additional cultures of sputum can be obtained for identification (Daley et al., 2020). Although AFB staining is widely used for diagnosis, it cannot differentiate between NTM and TB disease. Culture of NTM species takes time for reporting positivity. Thus, sensitive molecular tests like nucleic acid amplification tests (NAAT) like Xpert MTB/RIF (Cepheid, USA), Amplified *M. tuberculosis* direct test (Hologic, Inc., USA), Cobas TaqMan MTB test (Roche diagnostics, Switzerland), are used for differential diagnosis of NTM and tests like Genotype Mycobacterium CM (Hain life science GmbH, Germany), Reverse blot hybridization assay (REBA)-Myco-ID (YD Diagnostics, Yongin, Korea) are used for detecting NTM species.

Polymerase chain reaction-restriction fragment length polymorphism (PCR-RFLP) is a molecular method for NTM identification wherein a specific gene sequence is amplified by PCR followed by use of restriction enzymes to cut the DNA. The fragment patterns so obtained are analyzed for identifying mycobacterial species. It is cost-effective, has higher discriminatory power and is rapid, efficient when compared to the conventional methods. A study reported 100% sensitivity and specificity for identifying MTB and MAC species using this technique by amplifying the *hsp65* gene (Nour-Neamatollahie et al., 2017).

Real Time PCR (quantitative PCR or qPCR) is another useful technique for identifying NTM species wherein it amplifies specific DNA sequences in a sample and simultaneously measures the amount of DNA in real time. This is accomplished by the use of fluorescent probes or dyes that bind to the amplified DNA allowing the product to be detected and quantified. In the case of identification of NTM species by qPCR, the target gene is selected and then a multiplex PCR is done (amplifying multiple target genes for identifying different NTM species). By analyzing the amplification and melting curve patterns of various NTM species, specific NTM is identified. The High-resolution melting (HRM) analysis can be combined with qPCR for differentiating NTM species by analyzing the amplified DNA’s melting behavior (Peixoto et al., 2020).

Line probe assays (LPA) combine PCR amplification and reverse hybridization methods for identifying genetic markers associated with different NTM species. LPAs give faster results when compared to the traditional methods like culture and also have high sensitivity and specificity for identifying different NTM species. Some LPAs can also detect mutations associated with drug resistance in NTM. The above mentioned Genotype *Mycobacterium* CM (can detect *M. abscessus*, *M. chimaera* and differentiate it from other species) (Bruker Daltonics, Germany) and INNO-LiPA® Mycobacteria (Fujirebio, Belgium) can detect 16 mycobacteria species including *M. tuberculosis* complex (MTBC) and NTM species like MAC, *M. kansasii*, *M. xenopi*, *M. gordonae*, *M. avium*, *M. intracellulare* are the commercially available LPAs for NTM identification (Gürtler and Stanisich, 1996; Kirschner et al., 1993; Tevere et al., 1996).

Another technique used for NTM identification is Matrix assisted laser desorption ionization—time of flight (MALDI-TOF) mass spectrometry which identifies NTM based on unique spectral fingerprints produced by the extracted proteins. MALDI Biotyper (Bruker Daltonics, Germany) and VITEK MS (Biomérieux, France) systems are prominent players in catering to MALDI-TOF needs in this aspect. MALDI-TOF technique is simple, rapid and associated with low costs for consumables than conventional microbiological methods of identification (Alcaide et al., 2018). A study by Mediavilla-Gradolph et al. (2015) tested 66 mycobacterial isolates from various clinical isolates. The study found that MALDI-TOF was correct in 98.4% (65/66) of NTM isolated which included NTM species like *M. avium, M. intracellulare, M. abscessus, M. chelonae, M. fortuitum, M. mucogenicum, M. kansasii, M. scrofulaceum*. The study concluded that MALDI-TOF was a rapid and cost-effective method for identifying NTM.

Sequencing of selected genes like 16S RNA, hsp65, rpoB is a common method for identifying NTM. Multigene sequencing can help in enhancing the accuracy of identification in cases of mixed infections or closely related species. In these methods, PCR is initially done to amplify the gene/genes of interest which is then subjected to DNA sequencing (Kim and Shin, 2018).

The Whole Genome Sequencing (WGS) is a high-throughput technique which analyzes the entire genome of NTM. It provides detailed information about the species and sub-species hence it is very useful in NTM diagnosis. It can also help in identifying anti-microbial resistance genes for accurate prediction of drug resistance in NTM. Sequencing platforms like MiSeq, NovaSeq (Illumina technologies, USA); MinION (Oxford nanopore technologies, United Kingdom) use specialized software and databases for precise identification of NTM species (Dohál et al., 2021; Matsumoto and Nakamura, 2023).

Mycobacteria growth indicator tube (MGIT) is a liquid culture system used for detecting the growth of mycobacteria. MGIT-seq is a comprehensive and rapid method for extracting DNA from MGIT broth and using NGS to identify NTM species and detecting their drug resistance. This technique is useful in identifying NTM species and subspecies including the difficult to culture species. It also bypasses the need for subculturing which takes time. The study by Fukushima et al. (2023) observed that MGIT-seq gave 99.1% accuracy in NTM species level identification and 84.5% at the sub species level. Resistance of macrolide and amikacin were detected in 19.4 and 1.9% of MAC and *M. abscessus* isolates. The resistance so observed by MGIT-seq was found to be consistent with the traditional drug susceptibility tests. Thus MGIT-seq has been observed by the study to comprehensively aid in detection of NTM as well as identify resistance of NTM in a single test which the authors suggest will help in determining the treatment strategy by the clinicians.

Apart from these methods, emerging technologies like Clustered Regularly Interspaced Short Palindromic Repeat-associated proteins (CRISPR-CAS) particularly CRISPR-Cas12a based detection have gained popularity. Genome editing tool-based assays may be important in diagnosing NTMs because of problems with sensitivity and specificity that can result in false results, issues in capturing the genetic variability of NTM species, labor-intensive sample processing, related expenses, and costly laboratory equipment. The components of the CRISPR-based diagnostics are single-strand nucleic acid reporter molecules, a Cas effector, guide RNA (gRNA), the nucleic acid amplification mix, and CRISPR-Cas buffer. The target nucleotide sequence in these target gene assays is matched by gRNAs, which attract Cas proteins to the targeted site and cause nonspecific nucleotide cleavage (Dong et al., 2023; Li J. et al., 2024). A fluorescence-containing nucleotide reporter and a quencher are cleaved by the activated Cas proteins, generating a fluorescence signal allowing the detection of the target gene (Chen et al., 2018).

Recently, Compiro et al. (2025) developed a technology known as MyTRACK (Mycobacterium detection Tracing using Recombinase polymerase Amplification and CRISPR-Cas12a Kits) for detection and differentiation of *Mycobacterium tuberculosis complex* (MTBC) and other NTMs (Compiro et al., 2025). MyTRACK is a portable technique for identifying Mycobacterium species that requires only a few simple tools, such as a BluPAD Dual LED Blue/White Light Transilluminator that provides a fluorescent readout visible to the unaided eye and a heat box for RPA isothermal amplification, and it provides the results in 1 h (Compiro et al., 2025).

In order to amplify and identify DNA targets, this assay combines the CRISPR-Cas12a system and an isothermal amplification, known as Recombinase Polymerase Amplification (RPA) (Lobato and O’Sullivan, 2018). The target gene, RNA polymerase β subunit (rpoB), was chosen because of the substantial sequence variation it exhibits among various MTBC and NTM (Kim et al., 1999). The developed RPA primers and several Cas12a guide RNAs have been evaluated with each mycobacterial DNA in order to create the MyTRACK assay. Additionally, the improved MyTRACK’s limit of detection and cross-reactivity has been assessed. Clinical isolates and patient clinical specimens were used to compare MyTRACK’s performance to the culture method using LPA in mycobacterial detection (Compiro et al., 2025). The study reported that, with a threshold of identifying 1–100 copies/reaction of various targeted mycobacteria, the assay exhibits no cross-reactivity. The findings were in line with the culture method using LPA for clinical samples and showed 100% specificity and 92.59–100% sensitivity in clinical isolates (Compiro et al., 2025).

**Figure 1:**
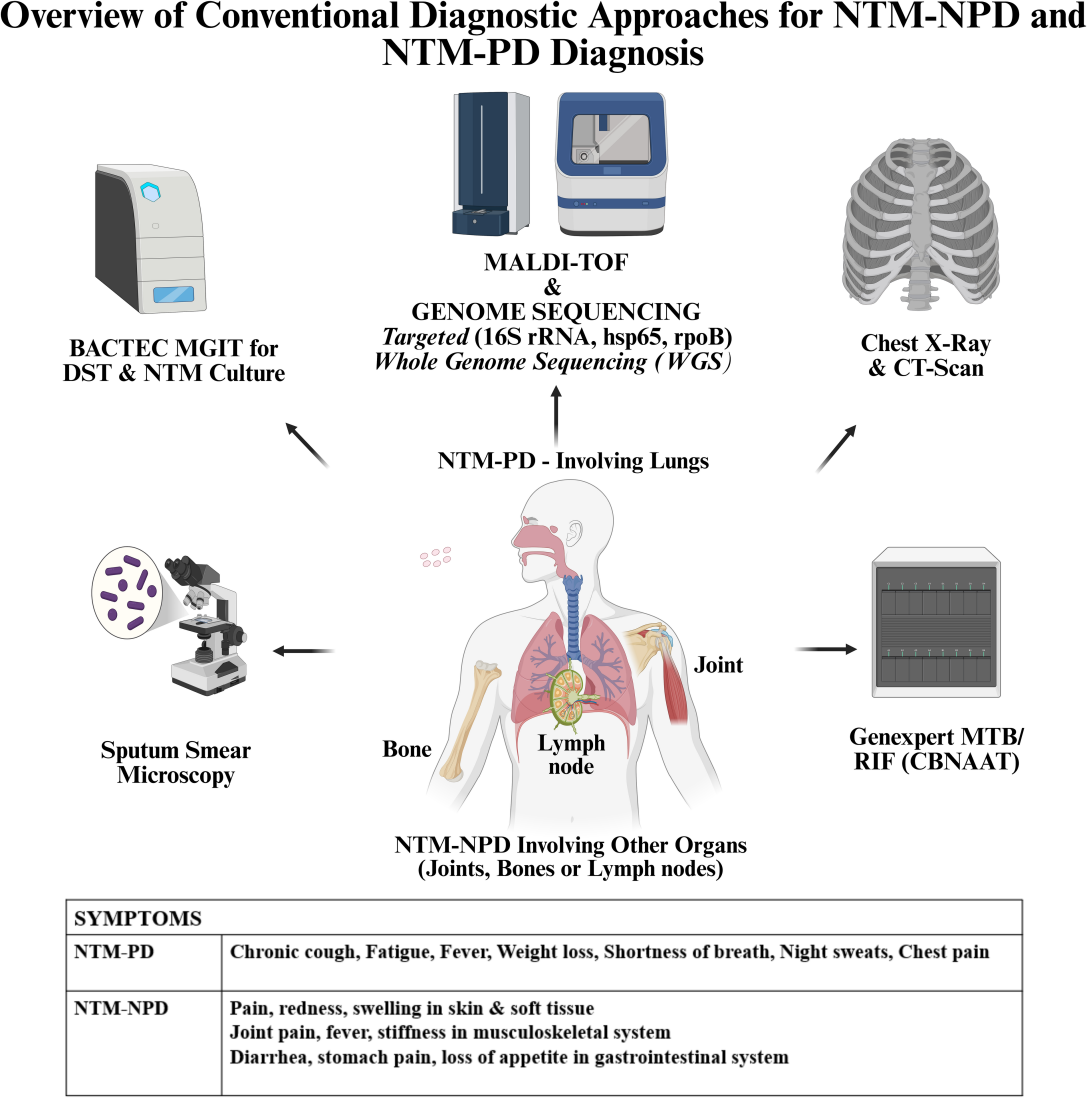
Overview of conventional diagnostic approaches for NTM-NPD and NTM-PD diagnosis.

Other conventional diagnostic methods for NTM include immunological, biochemical methods which are not sensitive and are time-consuming. Thus, methods like PCR, Real Time PCR, line probe hybridization assays, DNA sequencing, WGS, MALDI-TOF, CRISPR-Cas are more sensitive and have a greater potential in diagnosing NTM infections. The different diagnostic approaches used in NTM-NPD and NTM-PD diagnosis are given in Figure 1.

In case of involvement of organs other than the lungs in NTM (NTM-NPD), if fluid is accessible as in lymphadenitis, fine needle aspiration cytology (FNAC) is done and then the fluid is subjected to microscopy or culture for establishing the infection. In other cases wherein skin or other organs will be involved, excisional biopsy from that organ followed by microscopy for detecting AFB or culture may be the modality for diagnosis (Van Ingen, 2013). Tissue biopsy, aspirates and fluids are the different types of specimens used for NTM-NPD diagnosis. Sites for specimen collection include lymph nodes, bone (for osteomyelitis, arthritis), skin and other sterile body fluids. For suspected NTM-NPD, samples like skin biopsies, lymph node aspirates, bone biopsies, cerebrospinal fluid (CSF) may be collected for establishing diagnosis. In the case of disseminated infections, diagnosis is complex and will require a combination of tests from samples obtained from affected sites. Blood cultures are tested with MGIT for diagnosis in disseminated infections as the spread of mycobacteria happens from an initial location of infection to other organs or tissues through the bloodstream. Also, positive cultures from respiratory secretions, bone marrow, lymph nodes, liver, skin lesion, biopsies of the lung can also help in diagnosis of disseminated NTM infections. Examination of biopsies which reveal granulomatous inflammation may aid in NTM diagnosis even if AFB staining is negative. Imaging studies are also useful in the diagnosis of disseminated NTM infections or NTM-NPD like computed tomography (CT) scans of chest, magnetic resonance imaging (MRI) scans (in the case of bone or joint involvement), positron emission tomography (PET)/CT scans (for knowing metabolic activity in bones for bone marrow involvement).

Since NTM are ubiquitously present in the environment, their presence in the respiratory specimens does not necessarily indicate disease. Such overdiagnosis is a critical concern as per IDSA/ATS guidelines (Daley et al., 2020). The clinicians must avoid overdiagnosis based solely on positive molecular tests and must evaluate the results based on the clinical, microbiological and radiographic findings to arrive at a decision. Such overdiagnosis may lead to unnecessary treatments which may be having side effects and may cause harm.

Importantly, in laboratory settings NTM positivity can arise due to contamination rather than due to an infection because of the ubiquitous presence of NTM. During sample collection or processing, NTM can contaminate the medical tools and the reagents thereby contributing to false-positive results. Thus, implementing rigorous protocols during sample collection, processing and during storage may minimize contamination.

The ability to distinguish between NTM infection and colonization in non-sterile sites is crucial is initiating appropriate treatment. As per the IDSA/ATS guidelines, infection but not colonization requires antimicrobial treatment. According to the guidelines, positive culture alone does not signify infection but clinical, radiological and microbiological evidences are required for confirming the NTM disease by diagnosis (Daley et al., 2020).

### Treatment of NTM

The treatment for NTM infections is also equally challenging like diagnosis owing to the use of multiple drug combinations, prolonged administration of drugs, undesirable side effects. Thus, the decision to initiate the therapy occurs in consultation between the clinician and the patient with an explanation of potential risks and benefits the treatment may offer. In cases, where the patients present with moderate symptoms, and who are stable radiologically, they may be closely monitored and be made aware of the factors that may lead to disease exacerbation. Once the decision of treatment is decided upon, the patient is given drugs according to the inflicting NTM strain (as the NTM are genetically very diverse) as a single standard therapy would not be suitable for all the strains. The treatment is usually given for about 12 months and is continued even after the sputum shows negativity for AFB staining and/or absence of NTM colonies in culture in the case of NTM-PD (Daley et al., 2020). As extrapulmonary NTM (NTM-NPD) may involve skin and soft tissue infections, musculoskeletal infections, disseminated disease, lymphadenitis, the treatment for a specific NTM-NPD will be administered according to the disease and its severity. In the case of skin and soft tissue involvement, if the disease is mild, the treatment is for a period of 2–4 months and for 6 months if it will be severe and in the case of musculoskeletal disease, treatment is for a period of 6–12 months. For disseminated NTM disease, the treatment period is 6–12 months. The disease management in the case of NTM lymphadenitis is by surgical resection of the lymph nodes, antibiotic therapy or by an observational approach (Mi, 2019). During the course of treatment; sputum is frequently tested about 1–2 months if required in the case of NTM-PD (Daley et al., 2020). In the case of NTM-NPD, radiological imaging by CT scans are usually done before and after treatment and not during the treatment unless there will be lack of improvement. Also, cultures performed in specimens like synovial fluid, cerebrospinal fluid, tissue biopsies are useful for monitoring the response to treatment and for detecting drug resistance in the case of NTM-NPD. Frequency of repeating cultures for treatment monitoring response also depends on the clinical judgment. In the case of improvement of symptoms, cultures are repeated less frequently but if symptoms worsen, frequent cultures may be required. If the patient is immunocompromised, the frequency of doing cultures for monitoring may be increased. Apart from radiological and microbiological monitoring, clinical assessments of the patients are also done regularly to know the treatment response and for tackling adverse events which may occur due to the treatment (in both NTM-PD and NTM-NPD).

### Prognosis and treatment monitoring in NTM infections

The prognosis of NTM differs between individuals but untreated NTM infections can lead to severe illness and in some instances, death. In many cases, NTM is successfully treated while in some cases it can cause chronic health issues or it recurs even after treatment completion. The prognosis of NTM infections is dependent on various components like type of infecting NTM strain, location of the site of infection, underlying co-morbid conditions of the patient, age of the patient, severity and extent of the disease, response to treatment.

The degree of virulence exhibited by NTM and the treatment response varies between species. Infection with *M. abscessus* has been shown by earlier studies to have a poor prognosis when compared to infection with MAC (Jhun et al., 2020; Kotilainen et al., 2015). The patients with NTM-PD with the involvement of lungs had a higher mortality rate (Mourad et al., 2021) when compared to those with NTM-NPD wherein localized soft or skin tissues were involved (Aubry et al., 2002; Lindeboom et al., 2011). Patients with weak immune systems (HIV/AIDS) (Hu et al., 2022), lung pathologies (like bronchiectasis, chronic obstructive pulmonary disease [COPD]) (Fujita, 2025; Lee et al., 2024) and those with co-morbidities (like heart, liver disease) (Hwang et al., 2023) were reported to have a poor prognosis. Older individuals have a poor prognosis of NTM infections, specifically disseminated NTM infections (Chou et al., 2010). The mortality was found increased in patients aged more than 65 years, being male gender and those with high level of co-morbidities by a study (Andréjak et al., 2010). The prognosis was also worse in cases where the lung damage was extensive with cavitary lesions (Lin et al., 2025) and in individuals with severe form of bronchiectasis when compared to those who had a milder form of NTM disease. The prognosis also depends on the treatment effectiveness and the ability of the patient to tolerate antibiotic therapy and also their adherence to the treatment regimen (Pennington et al., 2021).

The monitoring of treatment of NTM infections is done currently by observing improvement in clinical symptoms, radiography (cavities, bronchiectasis, consolidations) and microbiologically by sputum AFB microscopy manifested by a decreased bacterial load in the case of NTM-PD, culture conversion (total absence of NTM) which is monitored for a prolonged period to confirm the success of treatment and prevention of relapse. In other cases where there will be extrapulmonary involvement (NTM-NPD), improvement in clinical symptoms will be used as a main yardstick for monitoring treatment response.

Thus, radiological and microbiological approaches for monitoring may be undertaken frequently wherever necessary for ensuring treatment efficacy and also for managing any potential complications. In the case of NTM lymphadenitis, ultrasound or CT scan may be used to monitor the response of the enlarged lymph nodes to treatment. For soft tissue and skin infections, imaging by monitoring may be necessary if the abscesses or infections are found in the deep tissues. The monitoring of musculoskeletal infections of NTM is done by performing MRI of the spine or affected bones or joints for knowing about the progression to osteomyelitis or the involvement of vertebra. Regular monitoring of NTM infections is essential due to the possibility of failure during treatment and relapse after the completion of treatment.

### Difficulties faced in treating NTM

The treatment of NTM infections is a challenge because of diversity of NTM species, acquired and intrinsic antibiotic resistance, requirement of long-term therapy with multiple drug regimens and the side effects which occur due to treatment. Apart from these factors, the formation of biofilms and the non-specific symptoms exhibited during NTM infections also pose difficulties during treatment.

Due to the diversity of NTM species which manifest difference in virulence, susceptibility to drugs, the diagnosis of the pathogen and choosing the effective treatment for a specific species becomes difficult. The NTM are inherently resistant to common antibiotics and also acquire resistance during treatment limiting the treatment options. Efficient treatment of NTM requires long course of treatment with many antibiotics (e.g., treatment for MAC involves 3 drug regimens and it is for about 18 months) and the side-effects of the drugs ultimately decreases the patient compliance to treatment (Conyers and Saunders, 2024).

The ability of NTM to form biofilms and their tough cell walls makes anti-microbial drugs difficult to penetrate their niche for killing them (Cano-Fernández and Esteban, 2024). Also, since they are ubiquitous in nature, it is challenging to ascertain whether their presence in a sample is mere colonization or infection. The symptoms manifested in NTM infections are non-specific like fatigue or chronic cough which mimics other disease conditions hence the diagnosis is delayed (Pennington et al., 2021). As said earlier, a single positive culture for NTM from a non-sterile respiratory source does not necessarily confirm their presence. The challenges in isolation and identifying NTM in diagnosis also lead to delay in treatment.

Owing to the lack of robust diagnostic and prognostic tools for the management of NTM infections, the search for efficient diagnosis and prognosis using genomics and other ‘omics’ is being widely explored. The developments and pitfalls in the genomic technologies and the lacunae as well as future scope of genomics in NTM diagnosis and prognosis and the utility of artificial intelligence (AI) in the advancement of genomics is further elaborated in this review.

### Genomics

The ‘Genomics’ has made leaps and bounds in progress and has now contributed to a greater understanding of the genome of many organisms within a short span of time. Undoubtedly, it has also helped in unraveling the genome of many mycobacterial species. The conserved regions in the genome are the main targets for species identification and diagnosis. Most of the species’ identification in NTM is done through nucleic acid sequencing. The conserved regions that are mainly targeted include 16s rDNA and hsp65. Also, 16S-23S internal transcribed spacer (ITS) located between the 16S and 23S ribosomal RNA genes which shows significant variation between different NTM strains is targeted as it is more helpful in species-level identification. Apart from these regions, other targeted regions in NTM include *rpoB, dnaJ, sodA, recA, secA1, gyrA/gyrB*.

A brief note about these targeted regions for the diagnosis of NTM infections by gene sequencing is given below.

## Gene sequencing studies on specific genes for NTM diagnosis

### 16S rDNA sequencing

The gene that encodes the smaller subunit of ribosomal RNA is 16S rDNA. This region is highly conserved and it contains variations that are genus or species-specific present in certain positions of the sequence which is useful for species identification. 16S rDNA sequencing method for NTM identification is more rapid and is more accurate on comparison with the standard methods of NTM diagnosis (Cloud et al., 2002; Hall et al., 2003; Holberg-Petersen et al., 1999; Patel et al., 2000). Another advantage of this method is its ability to provide taxonomic relatedness of species that are newly studied. For identifying already confirmed and novel mycobacterial species, a database named Microseq 16S rDNA bacterial identification system was developed by Applied biosystems but it does not include many mycobacterial species. Although the entire 16S rRNA region is about 1,500 bp, the Microseq 16S rDNA identifies a large portion of the hypervariable region (500 bp) within the 16S rDNA. Moreover, it does not include sequences of many mycobacterial species as it is intended for universal bacterial identification and hence the results show up discrepancies if mycobacterial identification is aimed for (Cloud et al., 2002; Patel et al., 2000).

Another database which contains sequences of mycobacterial genome is ‘Ribosomal differentiation of microorganisms’ (RIDOM). It is a freely available database that has data related to phenotypic and genotypic characteristics, references related to the species and clinical information apart from the sequence data (Harmsen et al., 2003).

Although 16S rDNA sequencing is conventionally used for identifying NTM, it was observed that it was unable to distinguish between *M. chimaera* and *M. intracellulare* wherein conserved gene sequences like rpoB, hsp65, 16S-23S ITS were used for circumventing the issue (Somoskovi and Salfinger, 2014). Similarly, 16S rDNA sequencing was found to have poor discriminatory value in distinguishing between *M. chelonae* and *M. abscessus* (Kirschner et al., 1992; Kusunoki and Ezaki, 1992). Although it has the demerit of less variation within a species and indistinguishable between certain mycobacterial species, it is cost-effective, rapid, accurate method and produces reproducible results from different laboratories and hence it is still being used in many settings.

### Hsp65 for the identification of NTM

The *hsp65* gene codes for the Hsp65 heat-shock protein having 65 kDa molecular weight and it is present in all mycobacteria and has more variability when compared to the 16S rRNA sequence. Hence it is more useful for identifying mycobacteria species that are genetically related. This sequence variation of hsp65 is used for diagnosis of rapidly growing mycobacteria (RGM) as well as slow growing mycobacteria (SGM) to the species level (Ringuet et al., 1999; Steingrube et al., 1995; Taylor et al., 1997; Telenti et al., 1993). For identifying NTM from different clinical specimens, hsp-65 PCR Restriction fragment length polymorphism Analysis (hsp65-PRA) is the widely used method as it is rapid and inexpensive for identifying mycobacterial species in a single experiment (Devallois et al., 1997; Saifi et al., 2013; Varma-Basil et al., 2013). The other method which is used for NTM diagnosis is the gene sequencing of hsp65 (Kapur et al., 1995; Pai et al., 1997; Pascapurnama et al., 2023). The disadvantages of hsp65-PRA are that the fragments that are of equal sizes are not differentiated well and it is also difficult to identify fragments that are small. Also, a nitrogenous base change at a single site may modify a restriction site which either appears or disappears which complicates the process. Another drawback of PRA is in the analysis of patterns wherein the calculated sizes differ from the sizes that are published. A study has shown that Gelcompar program can aid in interpretation of the results of PRA (Da Silva Rocha et al., 1999). As said previously, regarding the poor discrimination of *M. chelonae* and *M. abscessus* by 16s rDNA sequencing can be overcome by hsp65 sequencing as the hsp65 sequence of these species do not overlap and hence is useful for precise identification of these species (Sajduda et al., 2012). Despite its demerits, hsp65-PRA is fast, relatively cheap and accurate method for identifying NTM.

### 16S-23S internal transcribed spacer for NTM identification

The internal transcribed spacer (ITS) region is a region present in the ribosomal RNA (rRNA) gene that separates 16S and 23S rRNA genes. There is a single ITS reported in bacteria between the 16S and 23S rRNA genes. This region is known to possess highly hypervariable transcribed sequences and has many insertions and deletions and is useful in the discrimination of many bacterial species (Gürtler and Stanisich, 1996). The ITS region not only shows variation in sequence as well as length among different species but also within an organism at different rRNA gene loci. In a genome, many copies of the rRNA operon are present. Also, in the PCR based on 16S rDNA, the primers specific for the genus are separated by target sequence that is lengthy (greater than 500 bp) (De Beenhouwer et al., 1995; Kirschner et al., 1993; Tevere et al., 1996) but in the case of ITS-PCR, a smaller PCR product is obtained which leads to a sensitive and efficient amplification of the target. In many of the studies, amplification of the 16S-23S ITS is followed by sequencer based capillary gel electrophoresis (SCGE) for identification. This method was found to produce rapid and reproducible slow growing NTM (SG-NTM) species (e.g., *M. avium, M. intracellulare, M. gordonae, M. chimaera*) identification when compared to High pressure liquid chromatography (HPLC) or in-house PCR (Subedi et al., 2016).

This technique was also efficient in identifying rapidly growing mycobacteria (RGM) like *M. abscessus*, *M. massiliense* (*M. abscessus* subspecies *bolletii*) (Gray et al., 2014). Also, this method of ITS -SCGE can be completed within 1or 2 working days on comparison with agar-gel based techniques which involves more labor. When compared to the sequence based 16S rRNA technique, ITS-SCGE is less expensive as it does not involve full gene sequencing and relies on gel electrophoresis for DNA fragment separation. It can also identify novel species and also has the potential of reproducibility of results between the laboratories. Unique ITS sequences of *M. avium* complex have been used for defining intrasubspecific taxons and the subspecies defined using sequences were referred to as ‘sequevar’ (Frothingham and Wilson, 1993; De Smet et al., 1995). This technique (ITS-SCGE) also overcomes the discriminatory issues for the *M. chelonae* and *M. abscessus* species found in 16S rDNA sequencing based on a 3–4 base pair difference found in the 16S-23S ITS fragment rRNA of these species (Gray et al., 2014).

### rpoB for NTM identification

The rpoB gene of mycobacteria which is about 3,600 base pairs, codes for the beta subunit of RNA polymerase which plays an important role in RNA synthesis. The rpoB gene has also been utilized for detection of drug resistance in tuberculosis disease (Vareldzis et al., 1994). This gene is also useful for the identification of RGM (Adékambi et al., 2003) as well as *M. avium* complex species (Salah et al., 2008). In a study, the *rpoB* gene had the ability to discriminate between *M. kansasii* and *M. gastri* although 16S rRNA analysis was not effective in the discrimination (Kim and Shin, 2018). It has also been observed that this gene had the ability to identify mycobacteria belonging to SGM as well as RGM category by another study (De Zwaan et al., 2014). The demerit in rpoB sequencing is the non-availability of sequences of the uncommon NTMs in the GenBank database as observed for *M. heckeshornense, M. conspicuum, M. bohemicum* by a study (De Zwaan et al., 2014). Also, the other lacuna is the lack of quality assurance in public sequence databases thus there is a requirement for thorough investigation of results.

#### dnaJ

This gene is one of the members of the heat-shock protein 40 (Hsp40) family and it has been shown to be involved in regulation of the activity of sigma factor 32 (heat shock). Two genes that encode Hsp40 homologs in mycobacteria are *dnaJ1* (Rv0352) and *dnaJ2* (Rv2373c). Studies have shown that DnaJ is used as an alternative tool to identify the species of mycobacteria (Morita et al., 2004; Takewaki et al., 1993). It was also found that the mean sequence similarity of *dnaJ1* gene between species was significantly less (80.4%) and hence it had a higher discriminatory ability when compared to the 16S rDNA (96.6%), rpoB (91.3%) and hsp65 gene (91.1%) (Yamada-Noda et al., 2007).

#### sodA

Superoxide dismutase (SOD) is an enzyme that catalyzes the conversion of superoxide anion (free radicals) into hydrogen peroxide and molecular oxygen. Thus, this enzyme plays an essential role in protecting mycobacteria against the oxidative stress of the host cells. In mycobacteria, two types of SOD are found: SODA and SODC wherein SODA (Mn, Fe SOD) is one of the major extracellular proteins released in the culture medium encoded by *sodA* gene and SODC (Cu, Zn SOD) contributes to a smaller amount of the total SOD and is encoded by the *sodC* gene.

Genus-specific probes for SOD were found to recognize 23 of the 28 mycobacterial species and it did not cross-react with 96 of the non-mycobacterial species as observed by a study. The study also observed species specific variable regions within the SOD genes of NTM viz., *M. gordonae, M. fortuitum, M. kansasii, M. scrofulaceum, M. avium, M. intracellulare, M. simiae* (Zolg and Philippi-Schulz, 1994). The sodA gene sequencing along with ITS gene sequencing revealed the prevalence of *M. fortuitum* and *M. abscessus* by a study (Setiawan et al., 2024).

#### recA

The recA gene found in bacteria carries out the function of homologous recombination in DNA, repairs the damage in DNA, and induces the SOS response for repair (Miller and Kokjohn, 1990). But few studies have been done with respect to the recA gene of *Mycobacterium* species. In a study, the recA sequencing showed low degree of similarity between the mycobacterial species when compared to the 16SrRNA sequencing method. In the case of *M. gastri* and *M. kansasii*, a similarity of 96.2% was found and for *M. aurum* and *M. leprae* 75.7% similarity was observed (Blackwood et al., 2000). The study also highlights the demerit of recA sequencing wherein the investigators observed that the recA sequences related to MTB complex species like *M. tuberculosis*, *M. microti*, *M. bovis*, *M. africanum* were identical. Hence they propound that recA sequencing is not an ideal method for differentiating species of the MTB complex (Blackwood et al., 2000).

#### SecA1

The general secretory (sec) pathway is the protein export pathway which has been studied substantially in *Escherichia coli* (Mori and Ito, 2001). Homologous to the *E. coli* SecA protein, SecA1 is found in mycobacteria and is involved in the protein transport across the plasma membrane. It is a pre-protein translocase ATPase which aids in protein transport. Mycobacteria possess two SecA proteins: SecA1 is a housekeeping protein while SecA2 is non-essential (for growth in culture) secreted protein but does the function of exporting some proteins (Braunstein et al., 2001). The secA1 gene was amplified and sequenced from 29 mycobacterial species and 59 clinical isolates in a study wherein the sequence variability allowed for mycobacterial species differentiation (Zelazny et al., 2005). The sequence was also efficient in identifying clinical isolates. But, the sequences were not useful in identifying the MTB complex species (Zelazny et al., 2005).

#### gyrA/gyrB

The gyrA gene encodes for a subunit of DNA gyrase of mycobacteria which is involved in DNA replication, gene transcription and decatenation of DNA after replication. This enzyme is tetrameric made up of two A subunits and two B subunits encoded by *gyrA* and *gyrB* genes, respectively. If mutations occur in this gene, it leads to resistance against antibiotics which target DNA gyrase. The resistance to fluoroquinolones is because of alterations in DNA gyrase. This is due to changes in quinolone resistance determining regions (QRDR) in the A (region 67–106) and B subunits (region 426–464) of mycobacteria. The structures of both A and B subunits are highly conserved because of the essential function performed by this enzyme.

Thus, sequencing of this gene can help in identification of drug-resistant NTM. It has been shown by earlier studies that detection of missense mutations at the positions 83, 84 and 87 of gyrA was a quick and efficient method for detecting fluoroquinolone resistance in mycobacteria (Cambau and Jarlier, 1996; Kocagöz et al., 1996). A study by Bernard et al. (2014) showed that mutations in gyrA/gyrB were found in 92 isolates of MTB that were resistant to fluoroquinolones (Bernard et al., 2014). The other 70 isolates had mutations related to ofloxacin resistance. The 22 remaining isolates had mutation in neither gyrA nor gyrB. Contrarily, in another study, it was observed that there were no resistance related mutations observed in gyrA or gyrB genes of 105 MAC strains or MABC strains which included 72 isolates which were resistant to moxifloxacin, a fluoroquinolone (Kim et al., 2018). Monego et al. (2012) found that in ciprofloxacin-resistant *M. massiliense* isolates, the frequent point of mutations were observed in gyrA gene (Ala-90 → Val) and not in the gyrB gene (Monego et al., 2012).

## Next generation sequencing (NGS)

Next Generation Sequencing (NGS) also known as deep sequencing or massively parallel sequencing is a DNA sequencing technology that sequences millions of DNA sequences at the same time. It has revolutionized genomic research as it is rapid, cost-effective and a high throughput technique. It was developed based on the Sanger sequencing method and in the second-generation sequencing, massively parallel sequencing platforms like Illumina and Ion torrent have emerged as high throughput techniques. Both of these platforms employ the technique of sequencing-by-synthesis wherein incorporation of labeled nucleotides by DNA polymerase allows detection of bases during the addition. The advent of NGS in NTM detection has led to rapid and accurate identification of NTM species. It has aided in detecting organisms that are difficult to culture and in identification of drug-resistant mutations.

### Applications of NGS

#### Whole genome sequencing (WGS)

The WGS method overcomes the limitations of the conventional methods of diagnosis. It is fast, helps identify mixed infections (infection of an individual by many mycobacterial species), outbreak of NTM infections, efficiently detects drug resistance, aids in infection transmission pathway analysis. Although it is the preferred choice of technique for diagnosis by many labs because of its many valuable attributes, it suffers from many demerits. Its high cost and requirement for specialized labs and equipment limits its use in resource-limited countries. Also, most of the WGS technologies rely on the culture of the specific mycobacterial species (for obtaining sufficient pure DNA for analysis) which takes time and is not feasible in many settings. The interpretation of WGS data requires expertise and use of validated algorithms which is another challenge.

#### Study of whole genome of NTM for diagnosis

The genome of NTM is not constant and varies between species and has been found to range from 3.5 to 8 million base pairs when compared to *M. tuberculosis* whose genome size is approximately 4.4 million base pairs.

The WGS technology is efficient in diagnosing different NTM species. It can identify massive numbers of single nucleotide polymorphisms which is done by the modern NGS technology (Yoon et al., 2020).

The rise in NTM infections in immunocompromised individuals, the elderly, cystic fibrosis (CF) patients, and patients with non-CF chronic lung disease, asthma, and non-CF bronchiectasis, has impelled genomic studies to identify the factors that determine pathogenicity, transmission trends, emergence, and response (Lin et al., 2022). The availability and affordability of WGS has revolutionized bacterial evolution and phylogenomic research (Quainoo et al., 2017). Transmissibility, global evolution, and taxonomic puzzles have all started to be clarified by the WGS of NTM (Lin et al., 2022; Quainoo et al., 2017).

Even though WGS technology is not yet available in all clinical settings, its cost is steadily declining, and some benchmark mycobacteriological research facilities are using it as a standard procedure for mycobacteria identification and *M. tuberculosis* genotypic drug susceptibility testing (Katale et al., 2020; Yusoof et al., 2022; Zaporojan et al., 2024).

To develop new, trustworthy diagnostic tools for the biomedical field, WGS technologies offer the potential for taxonomic reconstruction and the identification of distinct gene families that exist within the genus Mycobacteria (Dohál et al., 2021). The WGS approach also enables physicians to comprehend the worldwide diversity of NTM species better and get around the drawbacks of the traditional tests used to diagnose mycobacterial infections (Dohál et al., 2021). Individual NTM species phylogenetic SNPs and all single nucleotide polymorphisms (SNPs) linked to resistance can be found using WGS (Khieu et al., 2021). WGS additionally helps in the diagnostic testing of multi-NTM infections linked to two or more specific species (Khieu et al., 2021).

Despite the remarkable accuracy of WGS, short-read, and shot-gun sequencing (almost 75–500 base pairs) has been used for the great majority of genomic analyses in recent years (Bouso and Planet, 2019). However, it rarely yields closed genomes (Bouso and Planet, 2019). The completeness of microbial genomes is, in fact, less than 10% of the total microbial genomes. Therefore, fragmented assemblies are problematic in comparative analyses because they can fail to resolve repetitive and G + C-rich regions, unlink gene clusters, ignore recombination, and neglect insertion and deletion elements (Bouso and Planet, 2019; Peona et al., 2020).

In comparison to the standard line probe assays, Quan et al., 2018, evaluated the effectiveness of WGS for NTM classification (Quan et al., 2018). Their findings supported the high degree of agreement between the individual techniques, with sequencing demonstrating the highest discriminatory power (Quan et al., 2018). This included identifying rare species, such as *M. ratisbonense* or *M. tomidae*, and preserving complex diversity, which is crucial within the *M. avium* and *M. abscessus* complexes (Quan et al., 2018). This is because mycobacterial species are more homologous than other bacterial species, and conventional assays only include a small number of amplified genes (Dohál et al., 2021). WGS has a somewhat extended wait time (3–15 days) than line probe assay, which is a drawback (Dohál et al., 2021). Similar to this, Yoon et al. (2020), showed that WGS and traditional molecular genetic techniques (direct sequencing and PCR) were 100% compliant in diagnosing *M. avium* subsp. *hominissuis*, *M. abscessus,* and *M. intracellulare* (Yoon et al., 2020).

Furthermore, WGS has shown itself to be an effective method for differentiating between reinfection and relapse of NTM which is primarily caused by *M. abscessus* or *M. ulcerans* (Chawla et al., 2022). WGS also makes it possible to identify NTM at the clone level, which enables the determination of the ongoing disease’s form (relapse/reinfection) (Chawla et al., 2022). Flohr et al., 2020, have used WGS to diagnose factitious disorders brought on by NTM that could be mistakenly identified as chronic or recurrent infections (Flohr et al., 2020). The high recurrence rate of certain NTM infections to 10–48% after successful treatment highlights the necessity of accurately defining the disease’s form (Lee B. Y. et al., 2015).

Bryant and coworkers isolated *M. abscessus* from cystic fibrosis patients, carried out WGS, and examined the phylogenetic relationships between these isolates (Bryant et al., 2016). Remarkably, authors found that isolates of *M. abscessus*, common throughout the world showed evidence of human-to-human transmission of the infection among cystic fibrosis patients (Bryant et al., 2016). In addition to this, in the cohort of pediatric cystic fibrosis patients, Harris and colleagues showed no evidence of patient-to-patient NTM transmission (Harris et al., 2014). However, NGS studies of NTM transmission have prompted doubts that NTM could spread among immunocompetent as well as immunosuppressed people (Yoon et al., 2020).

WGS has also been demonstrated to be beneficial in the treatment of multiple-NTM infections, whose diagnosis is essential for better understanding species diversity in different lung anatomical regions and for setting treatment regimens and outcome expectations (Solanki et al., 2022). As per the study of the genomic sequences of *M. abscessus* isolates, Shaw et al., 2019 demonstrated that Children with CF have a diversity of subpopulations of *M. abscessus* with varying antimicrobial resistance profiles (Shaw et al., 2019). Because, the authors confirmed a greater diversity of *M. abscessus* in pleural fluid, chest wounds, and lung samples than in sputum, the study’s findings were also crucial for the clinical assessment of the illness (Shaw et al., 2019).

Compared to traditional techniques, sequencing data offered a more thorough subspecies analysis in mixed cultures (Khieu et al., 2021). In a patient with a persistent pulmonary form of the disease, WGS also helped in identifying a high cross-species diversity of *M. avium* complexes (Operario et al., 2019). Since commercial probes only resolve *M. avium* species to the complex level, these results increase the therapeutic implications of WGS (Operario et al., 2019). Additionally, Greninger et al. (2015), performed WGS technology to identify a polymicrobial infection in a brain abscess caused by very unusual and quickly growing *M. immunogenum* and *M. llatzerense* (Greninger et al., 2015).

Furthermore, WGS has also helped to characterize the worldwide nosocomial infection outbreaks. Haller et al. (2016) reported the infection caused by *M. chimaera* in patients who had heart surgery (Haller et al., 2016). These infections typically manifest as disseminated infections, vascular graft infections, or endocarditis of prosthetic valves (Haller et al., 2016). Labuda et al., 2020 identified saline flushes as a cause of nosocomial bloodstream infections in oncology patients and characterized a novel species of fast-growing NTM (*Mycobacterium* FVL 201832) using the WGS technique (Labuda et al., 2020).

#### Targeted next generation sequencing (tNGS) for NTM diagnosis

The tNGS technology revolutionized genomics by rapid and cost-effective sequencing of large DNA samples. On the advancement of NGS, the sequencing efforts were focused on specific regions of interest which led to the developments in tNGS. In this method, the target region is enriched by using biotinylated oligonucleotides or probes that are complementary to the target regions (hybridization capture). These probes hybridize to the target DNA and the captured sequences are amplified and sequenced. The other method of enrichment uses PCR for amplifying regions of interest which are then sequenced.

When compared to WGS, tNGS narrows down on specific genomic regions of interest leading to in-depth analysis of genetic variations. Thus, it is a cost-efficient approach when compared to WGS. The tNGS is a rapid and comprehensive method for diagnosing NTM and for identifying drug resistance in NTM. It also has high specificity in detecting NTM species when compared to metagenomic NGS (mNGS) (discussed below). Some tNGS methods can analyze clinical samples directly thus obviating the need for culture of the specimens. It is efficient in detecting drug resistance which can help in changing the treatment regimens accordingly. Since it provides results fast, it can lead to earlier diagnosis and initiation of treatment. On the downside, the cost of tNGS is high when compared to the conventional methods of diagnosis. It requires bioinformatics tools and expertise for data analysis. Thus, despite its disadvantages, tNGS is an efficient tool in diagnosis of NTM and the associated resistance for management of NTM infections effectively.

In a study by Zhang et al. (2024b), the performance of tNGS was compared with Xpert MTB/RIF for identifying MTB in sputum specimens and clinical strains (Zhang et al., 2024a). The study observed that tNGS was more accurate and detected more drug resistance mutations which highlighted its value in TB drug resistance monitoring (Zhang et al., 2024a). In another study, the subspecies of *M. abscessus* was accurately identified using the Deeplex Myc-TB assay (multilocus, tNGS based kit developed by Genoscreen, Lille, France). It provided results faster (1–2 days) than WGS and had the ability to identify NTM from direct respiratory specimens and did not require culture of specimens. This assay targets hsp65 gene for Mycobacterial species identification and also regions from other 18 genes (including rpoB) for detecting drug resistance (Buckwalter et al., 2023).

He et al., designed specific probes for capturing NTM and MTB single nucleotide polymorphisms (SNPs) and 1,325 drug-resistant markers based on a TB mutation library for diagnosis and detection of MTB and NTM as well as their resistance profile. This approach which targeted specific regions was useful for accurate diagnosis of MTB and NTM (He et al., 2020).

#### mNGS for identification of NTM

When NGS began to pave way for analyzing microbial communities without the requirement for culture, it led to the development of metagenomics. The mNGS development is linked to NGS advancements which made it a high throughput sequencing technique with reduced time and cost. It is a valuable tool for identifying pathogens in various clinical samples like blood, respiratory samples, cerebrospinal fluid.

Although it has been widely used to diagnose infectious diseases, mNGS has not been used very often to diagnose NTM-PD (Wei et al., 2023). The mNGS analyzes all the DNA and RNA of samples for identifying the microorganisms. mNGS is an innovative technology that shows great promise for diagnosing infectious diseases. Unlike conventional microbial culture techniques, mNGS is a quick diagnostic tool. mNGS uses rigorous sequence read mapping, biological reference database construction, and nucleic acid sequencing in clinical samples to identify potential pathogens. The diagnostic ability is significantly enhanced by using this technology (Zhu et al., 2021).

The differential diagnosis of MTB and NTM from BALF sample analysis using mNGS was found to be highly accurate (Xu et al., 2022; Zhu et al., 2021). Xu et al. (2022) found that, mNGS was able to identify the 23 patients with NTM-PD. The diagnostic sensitivity of mNGS in conjunction with Xpert was the highest (100%, 31/31), and the sensitivities of mNGS, Xpert, T-SPOT, and acid-fast staining (AFS) in the immunosuppressed population were 93.5% (29/31), 80.6% (25/31), 48.4% (15/31), and 32.3% (10/31), respectively. In this aspect, the specificities of AFS were 40% (2/5), whereas those of Xpert and T-SPOT were both 100% (Xu et al., 2022).

Zhang et al. (2023) reported that patients with bronchiectasis were more prevalent in the NTM-PD group (*p* = 0.007) (Zhang et al., 2023). The NTM reads number was significantly higher in AFS-positive patients among mNGS-positive samples in the NTMPD group [61.50 vs. 15.50, *p* = 0.008]. The sensitivity of mNGS, on the other hand, was 90.2%, significantly higher than that of AFS (42.0%) and culture (77.0%) (*p* < 0.001). The mNGS had the same 100% specificity as traditional culture in identifying NTM. Further, authors also observed that mNGS had a receiver operating characteristic curve area under the curve of 0.951, which was higher than that of AFS (0.686) and culture (0.885). mNGS also detected other pulmonary pathogens in addition to NTM (Zhang et al., 2023).

Wei et al. (2023) found that in the diagnosis of NTMPD, mNGS had a sensitivity of 81.4%, which was greater than that of MGIT 960 (53.6%) (Wei et al., 2023). mNGS had a 97.8% specificity in diagnosing NTMPD, which is comparable to MGIT 960’s 100% specificity. When diagnosing NTM-PD, the combined sensitivity of mNGS and MGIT 960 was 91.8%. The sensitivity of mNGS was higher than that of MGIT 960 (*p* < 0.05) for sputum, pulmonary puncture tissue fluid, and bronchoalveolar lavage fluid (BALF), with respective values of 84.8, 80.6, and 77.5%. mNGS and MGIT 960 had respective Area under the curve (AUC) of 0.897 and 0.768. Sputum (0.870), pulmonary puncture tissue fluid (0.903), and BALF (0.916) had the highest AUC of mNGS (Wei et al., 2023).

Wang and Xing (2023) also found that the most frequent presentation with NTM was NTM-PD, though some patients also had extrapulmonary NTM infections (Wang and Xing, 2023). Six different NTM species were found by mNGS analysis, mainly MAC, *M. intracellulare*, and *M. abscessus*. All 22 of the available samples had negative results due to difficulties with conventional routine culture techniques. NTM was detected in five of the 10 patients who had quantitative PCR (qPCR) testing (Wang and Xing, 2023). In another study Wang et al. (2023) found that all of the mNGS results were positive, and six patients had qPCR analysis (Wang et al., 2023). The standard specifically mapped read numbers (SDSMRN) of NTM ranged from 1 to 766. It took 1–4 days from sample collection to NGS results. Eleven of their 12 patients (one of whom was lost to follow-up) had significant lesions absorption on their CT scans (Wang et al., 2023).

### Relevance of the conventional methods and different genomic approaches for NTM diagnosis

Although multiple genomic approaches and conventional methods for NTM diagnosis have been elaborated, the adoption of method/methods for diagnosis depends on the clinical presentation of the patient, suspected type of NTM and the availability of testing resources. When the patient presents with clinical symptoms for NTM-PD like cough, shortness of breath, sputum production, weight loss, fatigue and do not respond to standard antibiotics for bacteria, TB or NTM is suspected. The radiologic findings (X-rays, CT scans) of the patient are then relied upon (radiographic opacities, nodular or cavitary and high-resolution computed tomography (HRCT) scans show multifocal bronchiectasis with many small nodules) to assess the extent as well the pattern of lung involvement. Microbiologic tests like sputum culture are then recommended for culture and detection of NTM. When the growth of NTM is slow or difficult, molecular methods are adopted. Many laboratories have limited resources available which hinders timely diagnosis of NTM. This is challenging in regions where TB burden is high wherein the focus is on TB and NTM infections are neglected. The necessary equipment, expertise for culture of NTM species (slow growers) is not available in many labs which impact the NTM diagnosis. Molecular diagnostic techniques like PCR and mNGS which can rapidly identify NTM and differentiate between species are also not available in many settings. The delay in diagnosis of NTM in resource-limited settings can lead to disease progression and increase in morbidity. For accurate diagnosis and treatment of NTM, a combination of clinical presentation, radiological and microbiological test results are considered.

When traditional microbiological methods like AFB staining, culture yield slow (several weeks) or inconclusive results, molecular methods like PCR are preferred for. It yields fast results and can even be used for detecting NTM in tissue samples. It is also useful in smear negative cases with lesser bacterial load wherein small amount of nucleic acid from the fewer bacteria can be amplified for NTM detection. It has the ability to distinguish between MTB and NTM which can guide in administering appropriate treatment. The disadvantages of this molecular method include the cost (when compared to culture and AFB staining) and the presence of environmental contaminants leading to false-positives.

When conventional methods of diagnosis prove to be inconclusive or when detection of NTM needs to be done at species level or for detection of drug-resistant mutations, WGS is opted for. When specific gene sequencing (like 16S, hsp65, rpoB) also fails to provide a conclusive diagnosis, WGS is considered to be the efficient alternative. WGS also has the ability to identify mixed NTM infections which is challenging with other methods of diagnosis. But specific gene sequencing has the advantages of reduced cost, faster turnaround time for affordable results in identification and NTM differentiation. Specific gene sequencing is well established and has been validated for routine NTM diagnostics thus making it amenable for making clinical decision. Overall, WGS provides a comprehensive genomic information but specific gene sequencing provides a faster, cost-effective and targeted approach for NTM identification and management. The mNGS and tNGS are different applications of NGS wherein the former, sequences all the genetic material in a sample and the latter sequences specific targeted regions of a genome. The mNGS can help in detecting wide range of NTM species including those NTM which are less abundant and those that are difficult to be cultured. The mNGS has the additional advantage of detecting co-infection with fungi, viruses or other bacteria which can make treatment difficult and can worsen prognosis. When compared to tNGS, mNGS has a shorter turnaround time as tNGS involves sequencing of the targeted genes and their analysis which may take longer time. The mNGS has been shown to have improved sensitivity in NTM diagnosis when compared to the traditional culture methods or PCR methods in BAL, sputum, pulmonary tissue (Wei et al., 2023). The tNGS is more specific when compared to mNGS as it targets known pathogens and their genes. It is more cost-effective and has a standardized work-flow which makes it easy to implement and interpret the results. Thus, to summarize, both mNGS and tNGS are valuable tools for diagnosis of NTM, mNGS has a faster turnaround time, broder detection of pathogens, ability to identify drug resistance thus showing its potential in diagnosing complex NTM infections. The tNGS is preferred in settings where specificity and cost are of utmost importance. Different molecular methods and genomic techniques used in NTM diagnosis is given in Table 1.

**Table 1:** Molecular and genomic techniques used for the diagnosis of NTM.

S. No.	Name of the technology	Uses in diagnosis of NTM	Species	Strength	Weakness	Approximate cost of testing per sample (in USD)	References
1.	Microarray Technology	NTM species can be identified using microarray technology by analyzing their DNA sequences.	*M. intracellulare, M. chelonae, M. abscessus*	High throughput, High sensitivity and specificity, Rapid results	Cost, Complexity, limited use in the laboratories	100–300	Huang J. J. et al. (2020)
2.	Line Probe Assays	Reverse hybridization and nucleic acid amplification are used in this assay to identify NTM species based on variations in the 16S rRNA and 16S-23S rRNA regions, the 23S rRNA gene, or the 16S rRNA spacer region.	*M. avium* complex, *M. abscessus* complex, M. ratisbonense, M. tomidae, *M.chimaera*	High sensitivity and specificity, accurate species detection, Rapid results	Limited ability to identify mutations, Potential for indeterminate results, High cost	25–30	Kirschner et al. (1993), Gürtler and Stanisich (1996), Tevere et al. (1996), Gray et al. (2014) and Huang W. C. et al. (2020)
DNA sequencing methods—specific target gene sequencing							
3 (i).	16S rRNA Sequencing	This approach is frequently used to identify bacteria, however because of its high sequence similarity, it may not be able to differentiate between closely related NTM species.	*M. bovis, M. avium/intracellulare, M. scrofulaceum, M. terrae, M. gordonae, M. kansasii, M. xenopi, M. malmoense, M. fortuitum/chelonae*	Rapid and Precise genus level identification, Cost effective, Identification of new species	Potential for Sequencing Errors, Impending for false negatives, Database restrictions	20–173	Holberg-Petersen et al. (1999), Cloud et al. (2002). Hall et al. (2003) and Opperman et al. (2024)
3 (ii).	hsp65 Sequencing	The hsp65 gene, which codes for heat shock protein 65, can be sequenced to produce strong phylogenetic trees and be utilized for NTM speciation.	*M. intracellulare, M. kansasii, M. celatum, M. fortuitum, M. arcueilense, M. avium complex*	High accuracy, cost effectiveness, rapid identification and differentiation of species	Misinterpretation, False negatives, Database limitations	40–100	Devallois et al. (1997), Pai et al. (1997), Saifi et al. (2013), Varma-Basil et al. (2013), Scoleri et al. (2016) and Pascapurnama et al. (2023)
3 (iii).	rpoBSequencing	A more accurate technique for NTM speciation is to sequence the rpoB gene, which codes for the beta subunit of RNA polymerase. This is particularly true for rapidly growing mycobacteria (RGM) and the *Mycobacterium avium* complex.	*M. mucogenicum*, *M. smegmatis*, *M. porcinum, M. abscessus, M. mucogenicum*	Detection of diverse species, Valuable in epidemiological studies, Detection of drug resistance	High cost, False negative, Requires particular proficiency	48–174	Adékambi et al. (2003), Salah et al. (2008), De Zwaan et al. (2014) and Kim and Shin (2018)
3 (iv).	Multigene Sequencing	Multiple gene sequencing (e.g., 16S rRNA, rpoB, hsp65) when combined was found to increase the precision and resolution of NTM identification.	*M. chimaera, M. gordonae, M. colombiense, M. mageritense, M. persicum*	Precise Identification, Detection of new species, Detection of Mixed infections	High cost and complexity, Need for particular proficiency	100–400	Patel et al. (2000), Cloud et al. (2002), Kim and Shin (2018) and Kim et al. (2021)
4.	Repetitive sequence-based PCR (Rep-PCR)	This method distinguishes closely related species or strains by creating distinct genomic fingerprints from repetitive sequences in the NTM genome.	*M. avium subsp. Paratuberculosis, M. avium subsp avium, M. avium subsp., silvaticum, M. xenopi, M. gordonae, M. celatum, M. smegmatis, M. ulcerans, M. lentiflavum, M. kansasii*	High biased power, Reproducibility, Rapid identification, Differentiation of narrowly linked strains	Reliance on high-quality DNA, False positives, Lack of consistency	100–300	Zhang et al. (2024b)
5.	Polymerase chain reaction-restriction fragment length polymorphism (PCR-RFLP)	An important development in gene sequence analysis has been made with the successful use of PCR-RFLP in the identification of NTM. To create profiles specific to a species or strain, this technique combines PCR amplification, restriction enzyme digestion, and electrophoresis.	*M. abscessus, M. abscessus massiliense, M. abscessus bolletii*	Sensitivity and Specificity, Species detection and classification, Cost-Effectiveness	Limited Multiplexing, Dependence on Database, Potential for contamination	2–6	Zhang et al. (2024b)
6.	Two-step multiplex PCR (mPCR)	This method enables the quick identification of several NTM species by amplifying multiple target sequences at once.	*M. avium subsp. avium, M. intracellulare, M. avium subsp. Hominissuis, M. fortuitum, M. massiliense, M. abscessus, M. kansasii*	Differentiation of NTM species, Sensitivity and Specificity	High cost, Limited multiplexing ability, Contamination	100–300	Kim et al. (2021)
7.	Multiplex real-time PCR with high-resolution melting (HRM) method	By measuring the quantity of target DNA present in a sample, this technique enables the quick and accurate detection of NTM.	*M. kansasii, M. abscesses, M. avium, M. fortuitum*	High Sensitivity, Speed, and Specificity, cost effectiveness	Primer Design Dependence, False negatives, limitation in identification of species	200–400	Peixoto et al. (2020)
8.	Mycobacteria Growth Indicator Tube (MGIT)-seq	This method enables thorough identification and drug resistance detection of NTM by combining sequencing with MGIT (Mycobacteria Growth Indicator Tube) culture.	*M. avium* subsp. *Hominissuis, M. intracellulare* subsp. *intracellulare, M. intracellulare* subsp. *chimaera, M. abscessus* subsp. *abscessus, M. abscessus* subsp. *massiliense, M. kansasii, M. lentiflavum, M. peregrinum, M. fortuitum* subsp. *fortuitum, M. porcinum, M. paragordonae,M. gordonae, M. chelonae, M. szulgai*	Rapid Results, Drug Resistance Detection, Comprehensive identification	High cost, equipment requirement	21–32	Fukushima et al. (2023) and Wei et al. (2023)
Genome sequencing approaches							
9. (a)	Whole Genome Sequencing (WGS)	By using WGS approach, physicians can better grasp the worldwide diversity of NTM species and get beyond the drawbacks of the traditional tests used to diagnose mycobacterial diseases.	*M. abscessus* complex, *M. chimaera*	Comprehensive identification, Detection of drug resistance	High cost and availability, Data Interpretation, Standardization	100–500	Quainoo et al. (2017), Quan et al. (2018), Dohál et al. (2021), Solanki et al. (2022), Zaporojan et al. (2024) and Zhang et al. (2024b)
9. (b)	MinION Sequencing	Using real-time sequence output, this technology enables quick and thorough identification of NTM species, even at the subspecies level.	*M. botniense, M. talmoniae, M. shigaense; M. aquaticum, M. dioxanotrophicus, M. neumannii, M. lehmannii, M. gallinarum, M. grossiae*	Portable and relatively low cost, Rapid Identification, High sensitivity and specificity	High cost, Potential for errors, Concerns for specificity	0.10–0.50	Matsumoto and Nakamura (2023)
9. (c)	Targeted Next-Generation sequencing (tNGS)	By this method, NTM diagnosis is made directly from clinical samples with or without the culture of the samples. It was found to be accurate and useful in diagnosis and detection of resistance in NTM	*M. abscessus* subsp. *bolletii, M. abscessus* subsp. *abscessus, M. abscessus* subsp. *massiliense*	Rapid diagnosis, High sensitivity and specificity, Cost-Effectiveness	High Cost, Data analysis complexity and clinical interpretation	100–300	He et al. (2020), Buckwalter et al. (2023) and Zhang et al. (2024a)
9. (d)	Metagenomic Next-Generation Sequencing (mNGS)	This method offers a more comprehensive picture of the microbial population present in a sample and may be able to identify all potential pathogens, including NTM, impartially.	*M. avium complex, M. abscessus, M. occasionalis, M. wolframei, M. intracellulare, M. torulare*	Rapid diagnosis, Co-infections detection, Identify fast growing NTMs, Sensitivity and Accuracy	High cost, Limited consistency, False positives	300–500	Zhu et al. (2021), Wang and Xing (2023), Wang et al. (2023), Wei et al. (2023) and Zheng et al. (2024)
10.	Matrix assisted laser desorption ionization-time of flight (MALDI-TOF)	It is very useful for rapid and accurate identification of NTM. Can help in differentiating between clinically relevant NTM species from the environmental NTM contaminants.	MALDI Biotyper® (Bruker Daltonics, Germany) can identify 182 mycobacterial species of the current 201 known species. Vitek MS (Biomerieux, France) Advanced spectra classifier (ASC) can identify 39 mycobacterial species.	It exhibits accuracy, speed, cost effectiveness, ability to distinguish between infection and colonization, ability to detect mutations associated with drug resistance in NTM, can identify mixed infections	Challenges in species differentiation, difficulty in lysing the tough mycobacterial walls to extract proteins for analysis, in mixed cultures, it is problematic for accurately identifying NTM proteins from other bacterial proteins	1–4	Leyer et al. (2017), Mediavilla-Gradolph et al. (2015), Alcolea-Medina et al. (2019) and Luo et al. (2022)

A comprehensive comparison of conventional, molecular, genomic, and Artificial Intelligence (AI)-based diagnostic methods for NTM diagnosis is given in Table 2.

**Table 2:** Comparison of conventional, molecular, genomic, and Artificial Intelligence (AI)-based diagnostic methods for diagnosis and differentiation of NTMs.

Conventional methods	Molecular methods	Genomic methods	Artificial Intelligence (AI) based methods
Radiography methods CT or chest X-rays (Cao et al., 2021). Routine screening or follow-up investigations not cost-effective. Sputum cultures and AFB smear: Sputum AFB smear and culture. Drug susceptibility testing (DST) and genotypic identification depend on culture, the gold standard for laboratory validation of NTMs (Zaizen et al., 2022). Biopsy for AFB presence: It reveals granulomatous inflammation or AFB positivity (Zaizen et al., 2022). Biochemical Tests: Biochemical tests include reduction of niacin secretion, Arylsulfatase test, Nitrate reduction, Catalase activity, tween eighty hydrolysis, Citrate utilization, urease activity, reduction of tellurite for different NTM species (Bhalla et al., 2018). Time-consuming and less-accurate hence has become obsolete and it has been replaced with sensitive molecular tests Blood culture with MGIT: Culture in BD BACTEC™ MGIT™ is the technique used for disseminated NTM detection. Growth detection is done by BD BACTEC™ MGIT™ 960 Instrument (Ferraro et al., 2024).	Multiplex PCR: Amplifies several target genes at once Allows numerous NTM species detection in one experiment (Peixoto et al., 2020). Gene sequencing (rpoB, hsp65, 16S rRNA): The most specific species-level identification (Kim and Shin, 2018). PCR-RFLP: Very useful in distinction of NTM species that have distinct fragment patterns. It is quick, precise, and economical to identify NTMs (Nour-Neamatollahie et al., 2017). Repetitive element PCR (Rep-PCR): Helps distinguish between closely related strains Useful for strain typing, especially for non-mycobacteria that proliferate quickly, such as *M. abscessus* (Zhang et al., 2024a) Probe-based restriction analysis (PRA) or hsp65 PCR restriction enzyme analysis (PREA): Frequently used to compare restriction fragment patterns to identify species within NTM group (Sajduda et al., 2012). MALDI-TOF MS: It is a reliable test, but may have limitations in differentiating the closely related NTM species. It might not be able to distinguish between closely related NTM (Alcaide et al., 2018). Line Probe Assays (LPA): LPAs give faster results. High sensitivity and specificity for identifying different NTMs LPAs identify drug-resistant mutations in NTMs (Yoon et al., 2020). CRISPR-Cas12a: Genome editing tool-based assays may be important in diagnosing NTMs Cas proteins deliver nonspecific nucleotide cleavage to the intended location. Produces a fluorescent signal that makes the target gene detectable (Chen et al., 2018).	Whole-genome sequencing (WGS): It covers the entire genome of organisms Facilitates strain typing, gene discovery for antibiotic resistance, and epidemic research (Lin et al., 2022; Quainoo et al., 2017). Sequencing of specific genes: More reliable species-level identification Achieved through targeted sequencing of rpoB and hsp65 (Somoskovi and Salfinger, 2014). Metagenomic Next-Generation Sequencing (mNGS): mNGS, identifies every nucleic acid in a sample Allows for the identification of a wider variety of NTM species and mixed infection strains (Zhang et al., 2023). Targeted Next-Generation sequencing (tNGS): Narrows down on specific genomic regions of interest leading to in-depth analysis of genetic variations. Cost-efficient approach as well as rapid and comprehensive method (Buckwalter et al., 2023).	Radiomics: Extracts quantitative information for analysis from medical photos in a high-throughput manner. A “radiomic signature” for NTM diagnosis is created by extracting quantitative information from medical images, such as CT scans (Al Bulushi et al., 2022). Machine Learning: For developing prediction algorithms, machine learning can be a highly useful method. Framework with an AUC of 0.9 that was based on a large number of lesions for radiomics tasks (Han et al., 2025). A linear support vector machine (SVM) for machine learning tasks was used to evaluate the diagnostic significance of cavities and bronchiectasis in distinguishing NTMs from TB. Deep Learning: Deep neural networks have been created to analyze chest X-rays and differentiate between NTM-PD and TB. Finding insertions, deletions, and single nucleotide polymorphisms in the genome is made easier by deep learning algorithms. Automatically extracts relevant information from complicated patient data, such as medical pictures (Liu et al., 2023).

### Pitfalls in utilizing genomics for the diagnosis of NTM

Although genomics is a powerful tool in NTM diagnosis and is very advantageous in diagnosing NTM infections, there are many drawbacks of this technology which need to be addressed. One of the major drawbacks is the high cost which leads to limited accessibility of this technique in resource-limited settings with high-burden of cases. Expertise is required for performing genomic sequencing as well as the interpretation of results which is another key challenge in genomics. Genomics also cannot differentiate colonization from infection which is truer in the case of respiratory samples (wherein colonization is more common). In such cases, complementary testing methods like culture and phenotypic testing along with clinical information may help in confirming the presence of infection. Also, the protocols for data analysis and interpretation of genomic data are still evolving for enhancing the accuracy and reliability of genomics in NTM diagnosis. Thus, genomics although promising in NTM diagnosis also has limitations hence interpreting the genomic data should be done in conjunction with the clinical data for a better diagnosis.

So, for an accurate and effective diagnosis as well as management of NTM infections, a multi-disciplinary approach based on clinical assessment, conventional diagnostic methods based on culture, genomics would be required.

### AI in the diagnosis of NTM

In recent years, new possibilities have been seen for the timely diagnosis and treatment of TB and NTM due to the rapid advancement of AI and its extensive use in medical research. In addition to helping doctors raise diagnostic accuracy, the AI machine learning models has made it possible for them to identify patients at higher risk of infection early on and prevent infection (Khalifa and Albadawy, 2024a). AI can also help doctors create customized treatment plans for NTM-TB patients who have various ailments.

For diagnosing NTM disease, AI models like machine learning (ML) and deep learning (DL) are increasingly utilized. They have the ability to analyze data types like medical images (CT scans, X-rays), vast amounts of genomic data (WGS, tNGS), lab test results for assisting in NTM diagnosis. Thus, they have improved accuracy, speed on comparison with conventional time-consuming methods (Al-Antari, 2023; Jia et al., 2021). Common machine learning models include support vector machines (SVM), Random forests. Deep learning models include neural networks (which includes convolutional neural networks (CNN) for image-analysis) and deep neural networks (DNN) which have been developed for analyzing chest X-rays and for distinguishing between TB and NTM-PD (Liu et al., 2023).

### Utility of AI models in analyzing genomic data of NTM

The AI models help not only in NTM species identification but are also useful for identifying drug resistance mutations and for comprehending the phylogenetic relationship between the strains. AI can integrate the genomic data with other types of data like clinical data to give a clear picture of NTM infection and disease. AI algorithms specifically deep learning models may help in identifying insertions, deletions, single nucleotide polymorphisms in genome and can thus classify genetic variations. Such variant identification will be important for identifying NTM species, track outbreaks and identifying resistance mechanisms (Dias and Torkamani, 2019).

Training of AI models on genomic data related to drug susceptibility profiles may help predict the resistance to antibiotics. This in turn may help clinicians make informed decisions for providing effective therapy against the resistant strains (Liao et al., 2025). The AI algorithms can analyze large NTM genome datasets and reconstruct evolutionary relationships between different NTM strains. This may enhance the understanding of the origin and spread of NTM infections and identification of transmission sources (Jia et al., 2021).

Thus, AI by its ability to integrate genomic information with clinical and other requisite information can help in personalizing treatment strategies for NTM disease. By this way, effective and targeted therapies can be given to the patients based on their characteristics and the NTM isolate with which they are infected (Dias and Torkamani, 2019).

### Accessibility of AI tools in genomics—features and utility in clinical studies

AI tools leverage machine learning and deep learning algorithms for analyzing genomic data including WGS data to accelerate NTM diagnosis. The cost of AI tools used in NTM genomics depends on specific application, data requirements and the computational resources. Certain AI tools like GenoMycAnalyzer (Kim et al., 2024), SAM-TB (Yang et al., 2022), Nontuberculous Mycobacteria Database (NTM-DB) (Lu et al., 2025), Mykrobe (Hunt et al., 2019) and TB-Profiler are available free of cost and are useful in analyzing WGS data. Platform like Emedgene (Illumina Inc., USA) has features like explainable AI (XAI) and automation for streamlining variant interpretation and hence is a paid service. The basic AI tools (like chatbots) used in diagnosis of NTM genomic data costs around US $5,000–US $15,000 and in the case of advanced, custom-made solutions, the cost is high and exceeds US $200,000. The factors which influence the cost of the AI tool also depends on the complexity of the tool, scale of deployment, specific applications of the tool, data requirements, maintenance and technical support required for the tool.

The algorithms which incorporate deep learning will be more expensive for development and implementation. AI models for genomic based NTM diagnosis require large, high-quality datasets for training (Dias and Torkamani, 2019). Creation and curation of these datasets also adds to the cost. A multi-site health system which deploys comprehensive AI solutions would have costs exceeding US $75,000–US $100,000 or more per month. Using AI for diagnostics related to genomics may be costing less when compared to using AI for drug discovery and development in genomics indicating that the application may be influencing the cost. Training AI models on large datasets may require significant data storage and processing for which infrastructure will be required contributing to the increase in cost. Also, the maintenance, updates and technical support also add up to the cost.

### Technical requirements

AI based NTM diagnosis by genomics requires robust algorithms, high quality genomic data and efficient computational resources. Deep learning models like CNN and DNN are suitable for genomic sequence analysis, identifying NTM species and finding drug resistant markers. For training and deploying the AI models, large genomic databases and computational infrastructure are required.

Genomic data (WGS/tNGS/mNGS) of NTM for analysis should be of high-quality with accuracy and comprehensiveness. It should be having minimal sequencing errors for efficient downstream analysis. AI models should be trained and optimized with large sets of genomic data for reliability and accuracy. For analyzing large genomic data, significant computational power is required like access to high performance computing clusters or cloud-based resources (Schadt et al., 2010). For data pre-processing, analysis pipeline should be optimized for appropriate timely results. Integrating genomic data with clinical data like Electronic Health Records (EHR) and imaging data (CT) can efficiently aid in diagnosis of NTM. Development of AI models that are interpretable and explainable (Explainable AI or XAI) is essential for enhancing trust as well as for adopting it in clinical settings (Ali et al., 2023). Validation and evaluation (based on performance metrics like sensitivity, specificity, accuracy) of AI models in clinical trials will increase their accuracy and clinical utility.

### Difficulty in analysis of AI data and practical limitations

Several data analysis associated challenges for AI based genomics for NTM diagnosis include: difficulties in collection of large data sets, variability observed in NTM growth (RGM and SGM), requirement for specialized expertise in AI, genomics, bioinformatics, high-performance computing and molecular diagnosis.

Since NTM infections are rare, well-annotated datasets required for training AI models are not easily available. NTM diagnosis includes varied testing methods like culture, molecular testing, imaging modalities giving rise to different types of data and formats that need integration which is a difficult issue. Also, since NTM species are enormous (>200) they have different growth patterns and genetic characteristics hence efficient AI models which can handle this heterogeneity are required. As mentioned previously, due to their ubiquitous nature, the positivity due to NTM may not necessarily represent a true infection but may also be a transient colonization which is another limitation in data analysis and model development. Another impediment in data analysis is the rigorous validation of AI models which is required in clinical scenario for increasing their accuracy and reliability in the real-world settings. AI-based genomic analysis of WGS data requires specialized bioinformatics pipelines and expertise in phylogenetic and variant calling which is another issue that needs to be addressed. In clinical settings, interpretable AI models (like XAI) have gained traction over black box AI models wherein in the latter; the internal decision process is hidden or opaque thus making it difficult to understand how the outputs are arrived at (Schwartz et al., 2024). Other practical limitations include ethical and regulatory concerns related to privacy of patients, data security and responsible use of diagnostic tools (Cath, 2018). Thus, addressing these limitations through research, standardization of methods, meticulous consideration of ethical and regulatory implications is essential for gaining the maximum benefits from this technology.

### Benefits

Clinically, AI based genomics for NTM diagnosis carries benefits like improved accuracy and speed by analyzing vast amount of data and will be faster than conventional methods. AI algorithms if trained to distinguish between NTM species can help in guiding treatment decisions as NTM species respond differently to treatment. AI can aid in predicting treatment success as well as development of drug resistance which can help in devising appropriate treatment strategies for proper disease management (Elalouf et al., 2025). It can also minimize human error and possesses the potential to interpret complex data for efficient diagnosis (Khalifa and Albadawy, 2024b).

Although AI based genomics shows promise in diagnosis of NTM, its routine clinical use is under development and has many challenges as elaborated above. Overcoming the challenges with robust clinical validation in different clinical settings will be essential for implementing it in clinical studies.

### Studies on AI for NTM diagnosis

The study by Jia et al. (2021) combined comparative genomics with machine learning for selecting new diagnostic markers to precisely identify five common disease-causing NTMs viz., *M. kansasii*, *M. avium*, *M. intracellulare*, *M. chelonae*, *M. abscessus*. For identifying the five NTMs with high accuracy the study included a panel of six genes and two SNPs for identifying the NTM with 90% accuracy (Jia et al., 2021). This study observed that the panel with six genes was able to classify the species accurately. The two panels also were efficient in differentiating five NTMs from MTB (more than 99% accuracy). This study was able to afford insights into the identification of NTM pathogens which were closely related, through machine learning and genomic approach (Jia et al., 2021).

Another study explored the histopathological analysis of mycobacterial infection by analysis using artificial intelligence (Zaizen et al., 2022). The study used undetected AFB from pulmonary TB autopsy cases (two), biopsy cases (40) for training AI (convolutional neural network) to construct an AI for detecting AFB. Bronchoscopy was performed in 42 patients and they were evaluated using AI aided pathology in AFB detection. Among those with mycobacterial infections (*n* = 16), seven patients were diagnosed with TB and NTM infection. The AI-supported pathology was compared with bacteriologic diagnosis from the BAL fluid (Zaizen et al., 2022). Of the 16 patients who had mycobacterial infections, AI identified 11 patients to be positive. On considering mycobacterial infection, the AI-supported pathology was more sensitive when compared to the bacteriologic diagnosis (86% vs. 29%) by the study (Zaizen et al., 2022). The study also found that the specificity of AI-supported pathology was 100%. The authors conclude that AI-supported pathology may exhibit higher sensitivity in diagnosing AFB when compared to bacteriologic diagnosis for samples obtained by bronchoscopy (Zaizen et al., 2022).

A retrospective study by Doyle et al. (2020) was done to characterize pre-diagnostic features of NTM-LD patients and for assessing the utility of machine learning models to identify undiagnosed NTM-LD patients (Doyle et al., 2020). The NTM-PD patients who were diagnosed between 2003 and 2017 in primary or secondary care or by the record of their treatment regimen were considered for the study. Risk factors and treatment related information were obtained in the pre-diagnosis period. The study also included control population which was enriched by including at least one of the selected predictors of NTM-PD. This was done to make the predictive model focus on learning to distinguish between different types of illnesses rather than differentiate between healthy and ill patients (Doyle et al., 2020). The study included 741 NTM-LD and 112,784 control patients. The common pre-existing diagnoses for NTM-LD were found to be asthma, COPD and treatments were penicillin, macrolides and corticosteroids (inhaled) (Doyle et al., 2020). On comparison with random testing, machine learning improved NTM-LD detection by almost 1,000-fold with an AUC of 0.94 (Doyle et al., 2020). Thus, this study delineates the applicability of machine learning to be applied on primary care data for screening of undiagnosed NTM-LD patients. The prediction algorithm also showed that there were a considerable number of undiagnosed NTM-LD patients in the UK (Doyle et al., 2020).

Han et al., 2025 classified NTM using deep learning and machine learning to research datasets of radiomics. The authors created a radiomics framework based on numerous lesions for radiomics tasks, and it achieved an AUC of 0.9 (Han et al., 2025). The diagnostic importance of cavities and bronchiectasis in differentiating NTM from TB was assessed using a linear support vector machine (SVM) for machine learning tasks (Han et al., 2025). The bronchiectasis-based model achieved an AUC of 0.84 ± 0.06. Further, researchers used a 3D-ResNet model to perform binary classification between NTM and TB in deep learning tasks (Han et al., 2025). On the test and external validation sets, authors obtained AUCs of 0.86 and 0.78 (*p* < 0.05), accuracies of 0.83 and 0.69, specificities of 0.57 and 0.63, and sensitivities of 0.92 and 0.75, respectively (Han et al., 2025).

Computed tomography (CT) has become a vital diagnostic technique due to its quick imaging capabilities and high sensitivities for lung abnormalities. Although AI-assisted CT interpretation holds great promise for enhancing NTM identification, the rarity of the condition has limited databases, impeding development. However, to provide more trustworthy results for the early diagnosis of NTM and TB, Ying et al. (2022), used a deep learning model to classify CT scans as either NTM or TB. They then coupled these results with the results of each patient’s T-SPOT test (Ying et al., 2022). Using preliminary chest X-rays, Lee et al. (2022), created a DL model that can predict the midterm and long-term morbidity of NTM patients; prediction increases with the inclusion of clinical data (Lee et al., 2022).

In rural and resource-poor areas, creating an AI-based automated screening platform can boost the accuracy of disease diagnosis, raise hospital outpatient clinic efficiency, and drastically cut down on the time patients must wait to receive diagnostic reports (Li et al., 2023). According to research, the AI-assisted system outperformed the physician alone in terms of specificity, sensitivity, positive predictive value, diagnostic accuracy, and negative predictive value (NPV) (Cao et al., 2021; Li et al., 2023).

Current AI algorithms may increase the categorization accuracy for five types of PTB photographs by 71%, and pertinent AI systems have been created recently to help medical professionals improve the sensitivity and accuracy of CT diagnostics of PTB by interpreting chest CT scans (Gao et al., 2020). Furthermore, it has been proposed that AI is 1,000 times more effective than humans at automatically detecting pulmonary problems (Cao et al., 2021; Shucai et al., 2021). It is interesting to note that another study correlated the accuracy and sensitivity of physicians and AI in reading CT scans of PTB patients. It discovered that doctors’ readings were 93.8 and 92.8% accurate, respectively, while AI’s readings seemed 95.49 and 90.4% accurate (Shucai et al., 2021). Yuan et al., 2014 reported that nodules, cavities, tree-in-bud pattern, and pleural effusion were all substantially more common in PTB patients than in NTM patients. Bronchiectasis and cystic alterations were substantially more common in the 20 individuals with NTM lung disorders than in the PTB patients (20.0% vs. 4.0%; *p* = 0.034) (Yuan et al., 2014). Additionally, patients with NTM-lung disease were significantly more likely to have cystic abnormalities and bronchiectasis on their CT scans (OR, 33.04; 95% CI, 3.01–362.55) (Yuan et al., 2014).

Al Bulushi et al. (2022) shows that radiomics may accurately distinguish NTM from other causes of lymphadenopathy in children (Al Bulushi et al., 2022). The term “radiomics” describes the high-throughput extraction of quantitative features from medical images for analysis and clinical decision support, as well as the utilization of medical images as mineable data. With the use of these extracted attributes, machine learning can be a very effective technique for creating prediction algorithms. The goal of radiomics is to examine and extract a variety of intricate quantitative characteristics, many of which would not be visible or useful when relying solely on qualitative picture analysis (McCague et al., 2023). The accuracy, precision, and recall through this radiomics model were classified nodes as NTM, pyogenic, reactive, were 68, 72, and 70%, respectively. The AUC was 0.89 when comparing NTM to all other types of lymphadenopathies (Al Bulushi et al., 2022). Therefore, this strategy may serve as the foundation for a significant non-invasive clinical decision-making tool for these types of patients if it is confirmed in bigger cohorts. This could increase diagnostic precision and reduce the need for invasive procedures.

The test performance metrics of the key studies mentioned above are as follows: The study on machine learning for six diagnostic markers manifested an accuracy of 90% in NTM identification (disease causing NTM) and 99% accuracy in differentiating between NTM and MTB (Jia et al., 2021). In another study, AI supported pathology showed higher sensitivity in diagnosing AFB when compared to bacteriologic diagnosis (86% vs. 29%) (Zaizen et al., 2022). The use of machine learning in a retrospective study for diagnosing NTM infection using risk factors and treatment related information improved diagnosis 1,000-fold with an AUC of 0.94 (Doyle et al., 2020). Another study which used DL and ML on radiomics datasets gave an AUC of 0.9 (Han et al., 2025). Ying et al. (2022) used a DL model to classify CT scans as either NTM or TB. When these results (CT image analysis) were coupled with T SPOT test, the precision was improved in classifying NTM-PD and PTB. While T SPOT alone had an accuracy of 80.3%, when combined with DL it increased to 89.8%. The study by Shucai et al. (2021) showed that when compared to readings by physicians, the accuracy and sensitivity of detection by AI was 95.49 and 90.4% when compared to 93.8 and 92.8% by the physicians (Shucai et al., 2021).

Thus, the test metrics of the different studies involving AI and conventional testing methods suggest that AI (DL, ML models) is efficient in enhancing diagnosis by AFB microscopy (86% vs. 29%), CT scan image analysis (AUC 0.9) (Han et al., 2025). Also, the combination of diagnosis by CT image analysis (radiomics) along with interferon gamma release assay (IGRA) (T-SPOT TB) improved the accuracy of NTM diagnosis as shown by Ying et al. (2022). The enhanced accuracy and sensitivity by the AI models in radiomics surpassed the performance metrics of the physicians by many studies (Shucai et al., 2021). All of these study results point to the fact that AI is very promising and it can enhance the performance metrics of diagnostic tests for NTM diagnosis. The studies on NTM diagnosis by inclusion of trained AI models also must take into account that mere presence of NTM cannot be considered as a disease as it could mean colonization. Inclusion of such false positives may confound the analysis and hence this must be considered before embarking on forming a dataset.

Thus, AI will be advantageous in NTM diagnosis by increasing accuracy, making faster diagnosis and in predicting the clinical prognosis of NTM and in the differential diagnosis of NTM-PD from other lung diseases like TB. In the context of genomics, by leveraging machine learning and deep learning techniques, AI can aid in rapid NTM species identification, drug resistance prediction and also help in deciding on the course of treatment for successful therapy.

Although the AI platform looks promising in AI diagnosis it has limitations like the availability of datasets, training and validating AI models and integrating it into the clinical workflows, cost, expertise, ethical issues (related to patient’s data) and other demerits as described in the previous section.

### Validation and implementation of AI tools in clinical settings for NTM detection

Although research on many AI tools is being carried out for NTM diagnosis, the number of tools validated in clinical settings is less. Technologies like machine learning, deep learning have been utilized for medical imaging analysis and molecular diagnostics. The 3D ResNet model (a CNN) described in the previous section (Han et al., 2025) employs chest CT images for diagnosing NTM-PD from TB lung disease and has been validated by earlier studies (Han et al., 2025; Wang et al., 2021). But this model has also not been widely implemented for NTM diagnosis in clinical settings because it needs robust validation in diverse settings including countries with low resources and high TB burden. Also, the cost-effectiveness of the model also has an important role in wide spread implementation. If the models have the potential to show high accuracy in different populations, they should also effectively address other issues like algorithmic bias and data privacy.

In the case of AI tool-based diagnosis of NTM by genomics, as mentioned earlier due to the scarcity of publicly available genomic datasets (with high quality, comprehensiveness) for different NTM species needed for training AI models, broader validation and implementation is not possible. Another issue is the complexity of the genomic data and the similarity between different NTM species which hinders the development of accurate models. Thus, AI-based genomic tools are still in the research phase and the addressing of these challenges may aid further in validation and implementation in clinical settings.

## Perspectives and future directions

The future of NTM diagnostics looks more promising with the advent of new technologies and developments. The diagnostic tests for NTM in future should focus on accurate, rapid and methods that are not culture-dependent. This encompasses the currently used advanced molecular diagnostic tools and the efficient AI aided tools which are being explored for their utility.

The tNGS is preferred in many settings as it provides accurate and fast identification of NTM species at the sub-species level and for its cost-effectiveness when compared to the WGS. But when its cost becomes minimal (compared to conventional diagnostic methods) it may become an affordable technique in many resource-limited settings.

The potential of AI needs to be harnessed for early detection of NTM so as to make decisions for initiating appropriate treatment. The AI powered tools can be evaluated further for analyzing clinical, microbiological, radiological data to enhance the diagnostic accuracy and for predicting treatment outcomes.

Non-culture-based diagnostics include rapid diagnostic tests which are being developed and validated for early NTM diagnosis. This includes methods like NAATs or CRISPR-based blood test methods which are faster than the conventional culture methods. A study by Li L. et al. (2024) has demonstrated that their CRISPR MAC assay detected *M. avium complex* pulmonary disease (MAC-PD) with 97.6% sensitivity and specificity (Li L. et al., 2024). The study was done in MAC cell free DNA in serum samples. The study showed that the serum-based rapid assay was efficient for the detection of MAC infection and monitoring the response to treatment. Non-culture-based diagnostics also includes the advanced imaging technologies like HRCT scans which may help deduce patterns associated with NTM-PD disease with the help of AI in the future.

Although this review has focused mainly on the diagnostics of NTM from the genomic standpoint, the use of MALDI-TOF technique deserves special mention in NTM diagnostics. This technique analyzes the protein finger-prints of NTM compares it with commercial databases which have a number of mycobacterial spectra (e.g., reference spectra for 182 different mycobacterial species available in Bruker Daltonics platform MALDI Biotyper® HT mycobacteria module) and then provides accurate identification of NTM species. The turnaround time for this technique is few hours when compared to genomic methods which take more time. But, the cost of this technology has made it uncommon in many settings.

Although many technologies are being developed for early diagnosis of NTM and for monitoring treatment, still a profound understanding of NTM pathogenesis is required. This may help in delineating effective therapies for control of NTM, as treatment is also an essential part of NTM disease management. Also, the biofilm formation of NTM and their survival inside the cells need to be studied in-depth for the designing of effective therapies.
